# The Mechanism of Nucleophilic Substitution Reactions at an Iodine Center: Single-Well or Double-Well Potential Energy Surface?

**DOI:** 10.1021/acsphyschemau.6c00052

**Published:** 2026-07-18

**Authors:** Ádám Horváth, Zoltán Benkő

**Affiliations:** † Department of Inorganic and Analytical Chemistry, Faculty of Chemical Technology and Biotechnology, 61810 Budapest University of Technology and Economics, Műegyetem rkp. 3, H-1111 Budapest, Hungary; ‡ HUN-REN-BME Computation-Driven Chemistry Research Group, Műegyetem rkp. 3, H-1111 Budapest, Hungary

**Keywords:** Nucleophilic substitution, Addition−elimination, Iodine, Hypervalent iodine, DFT calculations

## Abstract

Nucleophilic substitution (S_N_) reactions belong to the most important transformations in both organic and inorganic chemistry. Although S_N_ reactivity is well-known for many main group elements, its extension to halogen centers is restricted to a few examples, and the mechanism of such reactions lacks fundamental understanding. Recent achievements initiated our investigations to decipher the mechanism of the substitution reactions between various amide ions and elemental I_2_. Here, we present that these reactions follow an addition–elimination pathway through a charge-transfer complex (single-well potential energy surface), opposing a classical bimolecular mechanism via a central transition state (double-well potential energy surface), as reported recently. In addition, inclusion of a continuum solvent model for a polar solvent (such as water) remarkably impacts the thermodynamic feasibility of these reactions. Uncovering the mechanism of these S_N_2 reactions is a key step to a general understanding of S_N_ reactions involving other attacking nucleophiles and to possible future extensions toward further halogens.

## Graphical abstract

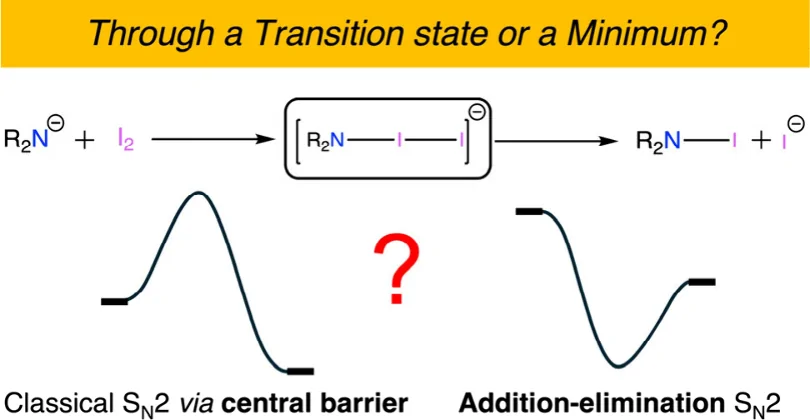

## Introduction

As a cornerstone of organic chemistry textbooks,^1–3^ nucleophilic substitution (S_N_) reactions are among the most fundamental elementary transformations.^4^ Following their first description more than a century ago,^5^ S_N_ reactions have found widespread applications in various fields of chemistry^6,7^ and even biology.^8–10^ Owing to its significance, this reaction is among the most studied chemical transformations,^3,11^ as demonstrated by the advanced experimental techniques^12,13^ and in-depth computational analyses^14^ that have been employed to clarify the mechanistic aspects.

For substitutions at an sp^3^-carbon center, two main mechanistic extremes are typically distinguished: (i) the stepwise monomolecular (S_N_1) and (ii) the concerted bimolecular (S_N_2) nucleophilic substitution;^4^ however, the separation is not necessarily straightforward.^15^ Generally, S_N_1 reactions are characteristic of tertiary carbon centers with good leaving groups and proceed through the formation of a cationic intermediate (in the first step), which is subsequently attacked by the nucleophile (Figure 1a). In contrast, S_N_2 reactions dominate at less hindered carbon centers (such as a methyl group). Specifically, canonical S_N_2 reactions pass through a single transition state that connects the two wells of the reactants and products (double-well potential energy surface) and leads to the stereospecific (so-called Walden) inversion of configuration at the attacked carbon center (Figure 1b).^4^

**Figure 1:**
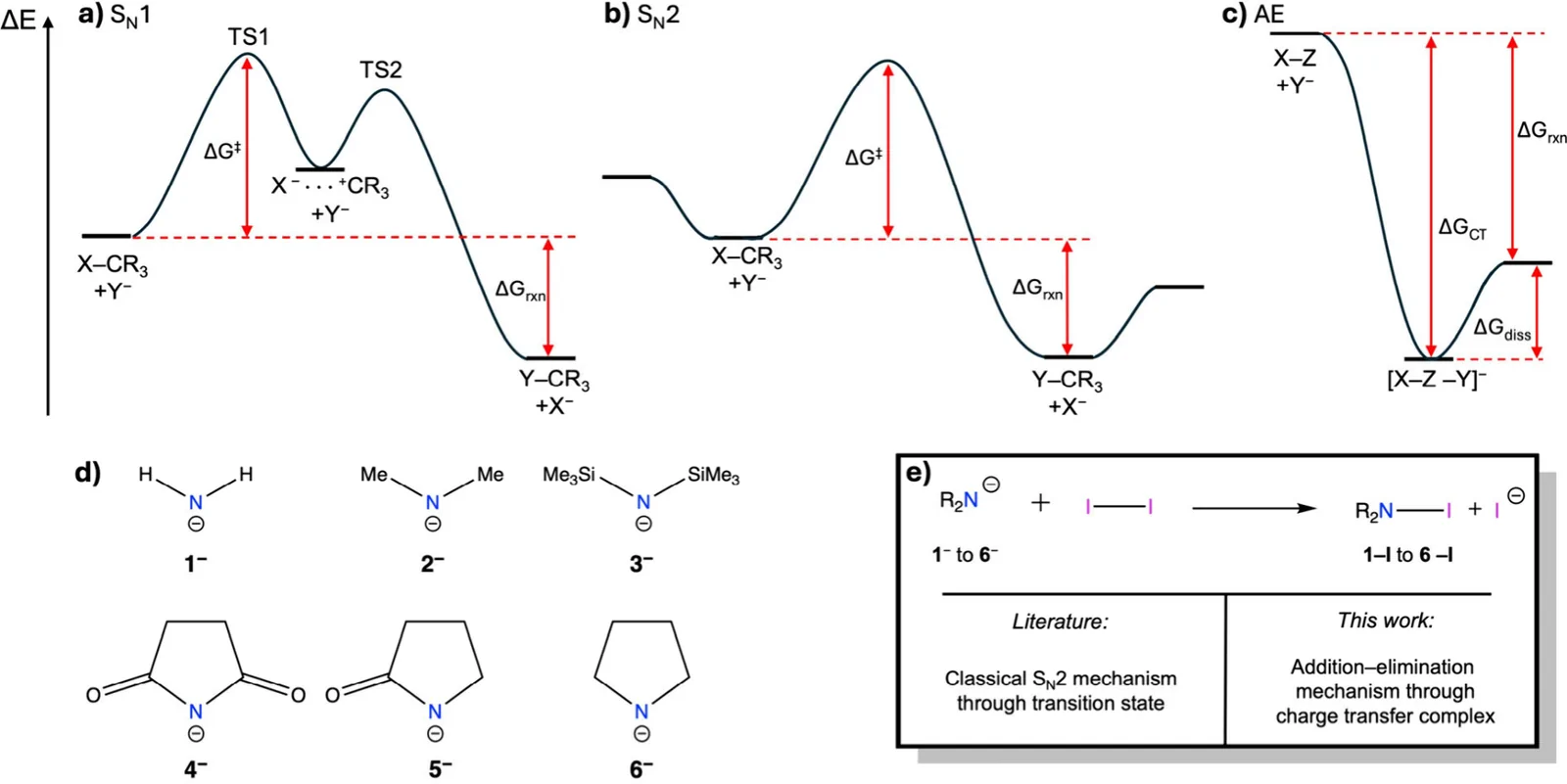
Schematic comparison of energy profiles of classical a) S_N_1 (typically R = alkyl), b) S_N_2 mechanism via a central barrier (double well, typically R = H), and c) S_N_2 addition–elimination (AE) mechanisms (single well), d) the investigated anions, and e) the substitution reactions considered in this work.

Beyond carbon, the S_N_2 reactivity at heavier main group centers such as P,^16–18^ Si,^19,20^ and S^21–23^ has also been extensively investigated over the past decades. Contrasting the classical S_N_2 reactions in organic chemistry, the substitutions at the elements in the third period or below commonly proceed through an addition–elimination (AE) sequence involving a hypervalent intermediate (charge-transfer complex) without a distinct transition state (Figure 1c).^4^ The corresponding potential energy surfaces (PES) are referred to as single-well.

Even though S_N_2-type reactions have been described for many main-group elements, substitutions at halogen centers are scarce. Early experimental evidence for the substitution reactions at an iodine center stems from the reaction of I_2_ with ammonia (NH_3_), whose hydrogens are replaced by iodine atoms.^24,25^ Similar behavior has been proposed for the pyridine + I_2_ ^26–28^ or PPh_3_ + I_2_ reactions.^29,30^ The possibility of an S_N_2-type reaction between X_2_ dihalogens (X = F, Cl, Br, I) and R_3_N amines (ammonia, trimethyl amine), or pyridine was computationally explored.^31^ These reactions, leading to the corresponding ion pairs through charge separation, proceed without a central S_N_2 barrier, but in an AE manner. Although most of these reactions are thermodynamically disfavored, an oriented external electric field can significantly affect the energetics of these processes.^31,32^ A variant of the classical S_N_2 reaction, in which the halogen is the main reaction center, is the so-called halogenophilic nucleophilic substitution (S_N_2X). In this case, the attack of the Nu^–^ nucleophile is proposed to happen on the X halogen center (instead of the carbon) to form the adduct Nu-X (assuming that X is in a +1 oxidation state) along with a carbanion. Then, in a subsequent step, the attack of the carbanion on the Nu moiety of Nu-X finalizes the reaction to form the classical substitution products without inversion. This reaction type was reported e.g. for specific α-halogeno esters^33–35^ or iodonitrobenzenes.^36^ Moreover, halocyclizations^37^ as well as iodochlorianations^38^ were described to followsimilar elementary steps.^39^ As an alternative to these pathways, a mechanism proceeding through single electron transfer (SET) has also been postulated.^40^

An illustrative model for nucleophilic substitutions at halogen centers hypothesizes that the attacked iodine center has an oxidation state of +1, and two, formally anionic groups exchange at I^+^ (X^–^ + [I^+^←Y^–^] **→** [X^–^→I^+^] + Y^–^), playing a role analogous to that of the central carbon in an S_N_2 reaction (Figure 1a).^31^

Recently, the reaction products of such reactions, exhibiting iodine in +1 oxidation state, have found prominent applications in electrochemical research, enabling the development of high-energy-density iodine-based batteries.^41–45^ In this context, Lee, Fan, and co-workers examined the reaction of I_2_ with several nitrogen-centered nucleophiles and proposed that the reactions proceed via a central transition state similarly to the classical S_N_2 reaction (Figure 1b).^46,47^ These results attracted our attention, as it markedly contrasts the typical addition–elimination-type S_N_2 reactions characteristic of heavier main group elements. In addition, the negative sign of the reported activation barrier seems unrealistic for a classical double-well potential curve.

In the present work, we demonstrate that nucleophilic substitutions by amide ions at iodine centers are correctly described as an AE-type S_N_2 reaction with single-well PES (Figure 1c). Moreover, we successfully extended the scope of this reactivity toward various other nucleophiles.

## Results and Discussion

To decipher the mechanism of the substitution reaction at an iodine center, we computationally examined the reaction between I_2_ and a diverse set of representative N-containing anions: the parent amide [NH_2_]^−^ (**1** ^**–**^), dimethyl amide [NMe_2_]^−^ (**2** ^**–**^), bis­(trimethylsilyl) amide [N­(SiMe_3_)_2_]^−^ (**3** ^**–**^), succinimidate (**4** ^**–**^), 2-pyrrolidonate (**5** ^**–**^), and pyrrolidinate (**6** ^**–**^) anions (Figure 1d). First, we conducted a thorough testing of different DFT functionals [ωB97X-D4, ωB97M-D4, M06–2X, B3LYP-D3­(BJ) and ωB2PLYP, combined with the def2-TZVPPD basis set; see Tables S1–S2], and all of these methods delivered generally similar results. For consistency, we selected the B3LYP-D3­(BJ)/def2-TZVPPD level of theory for geometry optimization, as the same functional was also applied in the report of Lee, Fan et al.^46^ Subsequently, on the optimized geometries, we carried out single point energy calculations at the state-of-the-art DLPNO–CCSD­(T)/def2-TZVPPD level of theory. In the following, we will focus on the energetic data obtained at the DLPNO–CCSD­(T)/def2-TZVPPD level of theory, unless otherwise stated.

We started to investigate the reactions depicted in Figure 2a in vacuum, analogously to the original calculations by Lee, Fan et al.^46^ Under these circumstances, our results unequivocally demonstrate that these reactions follow an addition–elimination-type mechanism (see Figure 2a), markedly contrasting the classical S_N_2 mechanism through a central barrier as recommended in that previous report (see above). First, I_2_ forms rather stable adducts, so-called charge-transfer (CT) complexes (abbreviated as [**1**-I_2_]^**–**^ to [**6**-I_2_]^**–**^), with the anions **1** ^**–**^ to **6** ^**–**^. Subsequently, the CT complexes dissociate to the final products, accompanied by moderately positive dissociation Gibbs free energies (ΔG_diss_, Table 1). These complexes were characterized as local minima on the potential energy surfaces instead of first-order saddle points corresponding to transition states. The formation Gibbs free energy of the charge-transfer complexes occupies a broad range (ΔG_CT_ = −71.0 to −22.6 kcal mol^–1^, Table 1). In contrast, the spectrum for ΔG_diss_ is considerably narrower (+4.4 to +19.1 kcal), but the actual values depend on the type of the anion: approximately ΔG_diss_ = +5 kcal mol^–1^ for amide anions **1** ^**–**^, **2** ^**–**^, and **6** ^**–**^ but more positive for the conjugation-stabilized silyl-amide **3** ^**–**^ (+12.6 kcal mol^–1^) as well as the imidate anions **4** ^**–**^ and **5** ^**–**^ (+19.1 and +12.8 kcal mol^–1^, respectively). Additionally, we also investigated a possible single electron transfer between the anions **1** ^**–**^-**6** ^**–**^ and I_2_ to form the corresponding **1** ^•^-**6** ^•^ radicals and an [I_2_]^•–^ radical anion. The resulting separated radicals are generally less stable than the CT adducts (see Table S6). As our ultimate goal was to study the nucleophilic substitution (S_N_) reaction, we always considered the closed-shell solution of the wavefunctions for further elucidation.

**Figure 2:**
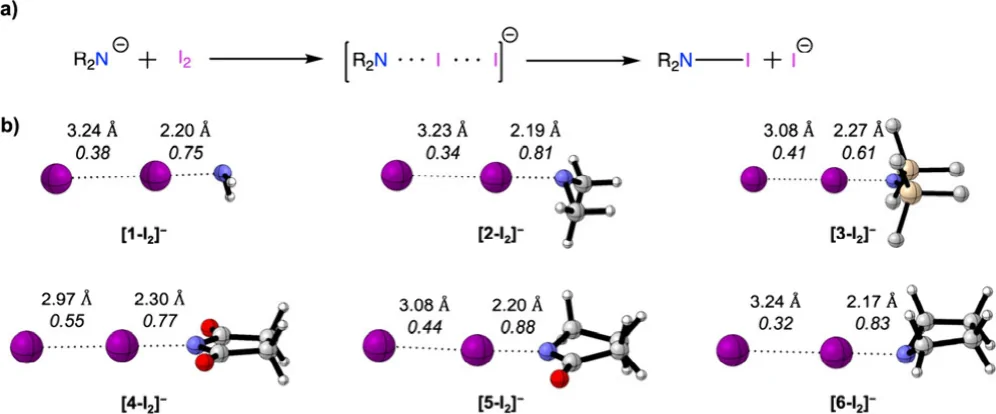
a) The uncovered reaction sequence. b) The structures of the formed charge-transfer (CT) complexes [R_2_N–I_2_]^**–**^ in vacuum, and the d­(N–I) and d­(I–I) interatomic distances (in Å, with normal letters) with the corresponding Mayer bond indices (in *italics*).

**Table 1:** Formation Gibbs Free Energies for the Investigated CT Complexes (ΔG_CT_: R_2_N^–^ + I_2_ → [R_2_N–I_2_]^−^), the Reaction Gibbs Free Energies for the Gross Reaction (ΔG_rxn_: R_2_N^–^ + I_2_ → R_2_N–I + I^–^), and for the Dissociation of the CT Complexes (ΔG_diss_: [R_2_N–I_2_]^−^ → R_2_N–I + I^–^)­^a^

	ΔG_CT_		ΔG_rxn_		ΔG_diss_	
Anion	vacuum	solvent	vacuum	solvent	vacuum	solvent
**1** ^–^	–71.0	–40.8	–65.5	–46.2	+5.5	–5.4
**2** ^–^	–64.6	–44.8	–59.4	–50.3	+5.2	–5.5
**3** ^–^	–32.0	–27.0	–19.4	–27.6	+12.6	–0.6
**4** ^–^	–22.6	–2.1	–3.5	–4.4	+19.1	–2.3
**5** ^–^	–36.3	–12.2	–23.5	–17.5	+12.8	–5.3
**6** ^–^	–61.1	–44.6	–56.7	–50.3	+4.4	–5.7

a The values were calculated both in vacuum and using the SMD implicit solvation model and are given in kcal mol^–1^.

Besides the thermodynamic aspects, the structures of the CT complexes are also influenced by the nature of the anion. The N···I and I···I interatomic distances [d­(N–I) and d­(I–I), respectively] in the CT complexes change remarkably upon substitution (see Figure 2): The d­(I–I) distance increases significantlyfrom 2.97 Å to 3.24 Å (in the **4** ^**–**^, **5** ^**–**^, **3** ^**–**^, **1** ^**–**^, **2** ^**–**^, **6** ^**–**^ order, versus 2.69 Å in I_2_), whereas the d­(N–I) distance moderately decreases from 2.30 Å to 2.17 Å (in a similar order). For comparison, the shortest I···I distance (2.89 Å) in [**4**-I_2_]^**–**^ is close to the corresponding distances in the I_3_^–^ anion (2.99 Å). Interestingly, the shortest N···I distance (2.17 Å) in [**3**-I_2_]^**–**^ approaches those in the R_2_N–I iodo-amine (or amide) products involved in this study (2.03–2.10 Å, Figure S1). To gain more insights into the bonding situations, we carried out Bader (atoms-in-molecules) analyses of the electron density using the DFT wavefunction of the CT complexes (Table S10), which delivered bond critical points between the N and I as well as between the two I centers for each of the CT adducts. Generally, both the N···I and I···I interactions exhibit positive Laplacian (∇^2^ρ) values alongside negative total energy densities (H) in the bond critical points, suggesting the presence of a dative bond (closed shell interaction).^48^ Agreeing with the calculated Mayer bond orders and the change in the interatomic distances (Figure 2), the N···I interactions have a higher covalent character than the I···I interactions in all the cases. Nevertheless, all these data suggest that the metrical parameters of the investigated CT complexes encompass a considerably wide range: As ΔG_CT_ becomes more negative, the I···I interaction weakens, and parallelly the N···I interaction gets stronger. Therefore, the three most stable CT complexes, namely, **3** ^**–**^, **4** ^**–**^, and **5** ^**–**^ may be described with a [R_2_N···I···I]^−^ structure that exhibits three-center-four-electron (3c-4e) bonds (also these I···I interactions are similar to those in I_3_^–^, Table S10).^49^ Conversely, the remaining adducts, with the most substantial ΔG_CT_, can be better regarded as weakly bound complexes between iodo-amines/amides and an I^–^ anion held together by a so-called halogen bond (σ-hole interaction; i.e., the theoretical limit of [R_2_N–I···I]^−^).^50,51^ Moreover, the lengths of the N–I bonds formed in the R_2_N–I products are in line with the formation energy of the corresponding CT complex. Indeed, the lowest ΔG_CT_ value is obtained for the anion whose reaction leads to the product with the shortest N–I bond.

Next, we aimed to elucidate the precise mechanism of these reactions at the molecular level and attempted to determine the transition states (TS) describing the formation of the above complexes and their potential dissociation to R_2_N–I and I^–^. Alternatively, we used the Nudged Elastic Band (NEB) method to locate possible transition states (see SI for details). However, all our efforts to locate such transition states remained unsuccessful. Therefore, we carried out one-dimensional (1D) relaxed scan calculations in the gas phase (Figure 3a-c), which were initiated from the CT complexes by varying either the N···I or I···I distance. Consistently, single-point DLPNO–CCSD­(T) calculations verify these DFT results. Because of the high similarity of certain data sets, we focus here on the experimentally most relevant anions **4** ^**–**^, **5** ^**–**^, and **6** ^**–**^,^46^ but all the remaining examples are provided in Figures S4–S5.

**Figure 3:**
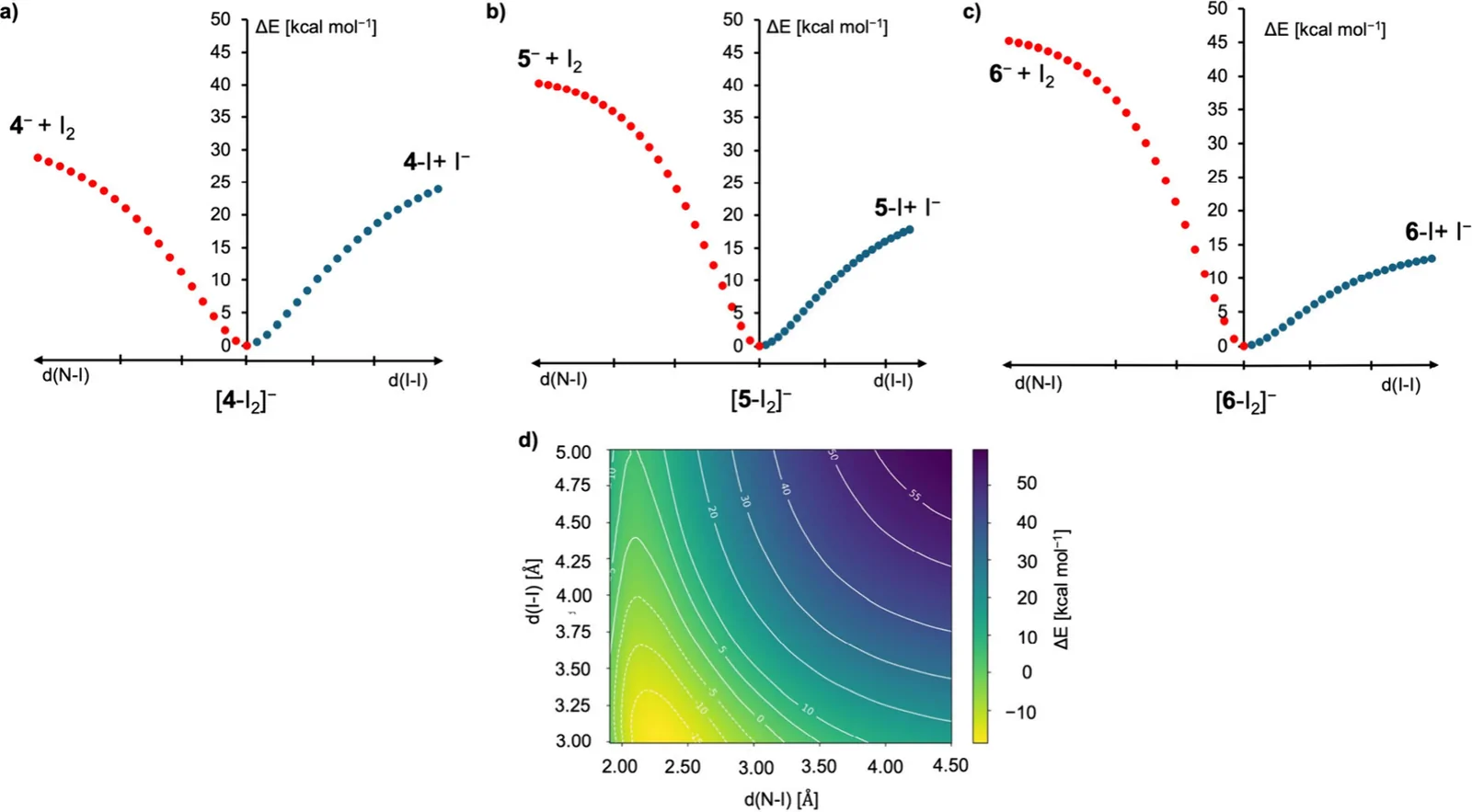
Relaxed 1D-scan calculations started from the CT adducts a) [**4**-I_2_]^**–**^, b) [**5**-I_2_]^**–**^, and c) [**6**-I_2_]^**–**^. The right and left directions along the horizontal axis represent increasing I–I and N–I distances, respectively. d) Systematic exploration of the two-dimensional (2D) potential energy surface (PES) of the **4** ^**–**^ + I_2_ system. The minimum around d­(N–I) = 2.3 A corresponds to the CT complex [**4**-I_2_]^**–**^.

In all cases, the 1D cross-section of the potential energy surface (PES) exhibits a single minimum, corresponding to the CT complexes. Besides these, no local extrema could be located, unambiguously proving the absence of a transition state or possible further intermediate(s) along the reaction coordinate. To explore the PES in more detail, we also performed an extensive two-dimensional (2D) scan, starting from the CT complex [**4**-I_2_]^−^ (Figure 3d). Importantly, no sign of additional stationary points could be located on the 2D-screening, except for the local minimum corresponding to the [**4**-I_2_]^−^ complex. The outcome of this 2D-scan perfectly agrees with the conclusion based solely on the 1D-scans (Figure 3a).

The presented results together outline a bimolecular substitution pathway for all investigated reactions. Importantly, all these reactions follow an addition–elimination type mechanism without any distinct activation barrier, resembling the behavior of other elements below the second period (such as P or S).^23,52,53^ Beyond doubt, our results contrast the S_N_2 mechanism proceeding via a central barrier (such as in classical organic S_N_2 reactions), as recently proposed for the same reactions by Lee, Fan et al.^46,47^

At this point, we need to clarify a further shortcoming of the previously reported model.^46^ Although the studied S_N_2 reactions, R_2_N^–^ + I_2_ → R_2_N–I + I^–^, are overall exergonic (see ΔG_rxn_ in Table 1 and Figure 3a-c), the separated products (R_2_N–I + I^–^) lie at significantly higher Gibbs free energies (and also in terms of electronic energies, see Figure 3) compared to the corresponding [R_2_N–I_2_]^−^ CT complex intermediates. This observation means that the dissociation of the complexes is thermodynamically disfavored (endergonic by ΔG_diss_ = +4.4 to +19.1 kcal mol^–1^, see Table 1). Searching for a possible explanation for these non-negligible, positive dissociation Gibbs free energies, we hypothesize that the gas phase calculations are unsuitable for precisely describing the energetics in the solution environment employed in the experiments. Therefore, we scrutinized the thermodynamic feasibility of the above AE-type S_N_ reactions employing implicit water solvation to mimic the experiments, which were conducted in aqueous solutions.^46^

Applying the SMD (solvation model based on density) parametrization, the formation of the CT adducts becomes significantly less exergonic (see the *solvent* columns in Table 1) in all cases, and ΔG_CT_ still encompasses a wide range between –44.8 and –2.1 kcal mol^–1^. Since the inclusion of solvent effects reduces the overstabilization of the CT complexes, the overall thermodynamic driving forces of the substitution reactions (ΔG_rxn_) change substantially compared to those obtained in the gas-phase (Table 1). For anions **1** ^**–**^ **-6** ^**–**^, the formation of the separated final products (the corresponding R_2_N–I and an I^–^ anion) from the CT complexes becomes thermodynamically favored as indicated by the negative ΔG_diss_ values (Table 1). Moreover, we conducted 1D-relaxed-scan calculations employing an implicit solvent model (Figure S6). These results qualitatively resemble those of the gas phase simulations, meaning that again no local maxima along the reaction coordinate could be identified for any of the investigated reactions, proving the absence of a central barrier. Besides SMD, for anions **4** ^**–**^, **5** ^**–**^ and **6** ^**–**^, we also assessed the effect of the CPCM (conductor-like polarizable continuum model) parametrization at the DFT level. The CPCM model yields somewhat more positive ΔG_CT_ and ΔG_rxn_ values compared to the SMD data, while the ΔG_diss_ values remain similar (Tables S3–S4). Moreover, we evaluated two additional solvents with different characteristics, namely acetonitrile and dimethyl sulfoxide. According to the SMD and CPCM calculations, both solvents show similarity to water, regarding the ΔG_CT_, ΔG_rxn_ and ΔG_diss_ energies (Tables S3–S4).

Finally, we aimed to identify the electronic properties of the attacking anions that primarily control the stability of the CT adducts. We have found that ΔG_CT_ becomes more negative with the destabilization of the HOMO (increasing ε_HOMO_) of the anions, showing a linear trend (see Figure 4). Notably, a similarly good linear correlation was obtained between ΔG_CT_ and the proton affinity across the series of N-centered anions involved in our study (Figure S7). These relationships highlight the leading impact of the nucleophilicity of the attacking anions on the formation of the CT complexes. Additionally, the gross reaction Gibbs free energies ΔG_rxn_ leading to the separated products (R_2_N^–^ + I_2_ → R_2_N–I + I^–^, see also Table 1) also follow a similar tendency as the ΔG_CT_ values (Figure S8). The above trends allow us to conclude that both the HOMO energy and the proton affinity of the anion markedly affect the stability of the CT complexes, and even more importantly, the thermodynamic feasibility of the substitution reaction.

**Figure 4:**
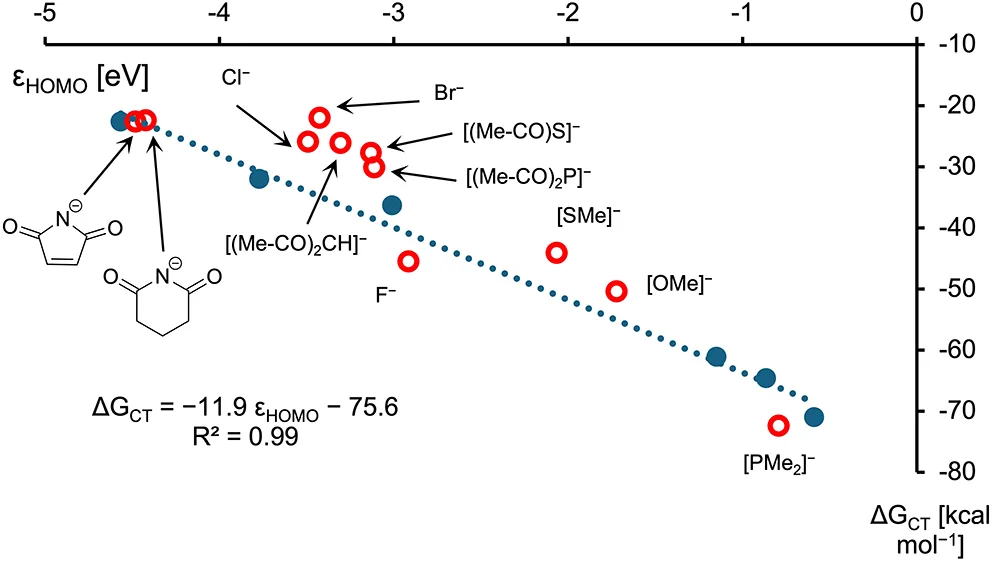
Linear trend between ΔG_CT_ in vacuum and the HOMO energy of the anions. The blue dots stand for **1** ^**–**^–**6** ^**–**^ anions, and the red, unfilled dots stand for additional nucleophiles (see text). The blue trendline was fitted on the set of anions **1** ^**–**^-**6** ^**–**^ (R^2^ = 0.99, Figure 1d).

Building on these observations, we hypothesize that ε_HOMO_ may be applied to estimate the thermodynamic feasibility of substitution reactions with further anions, possibly aiding the design of future experiments. To demonstrate the predictive value as well as to probe the generality of the mechanism across further anionic nucleophiles, we extended the scope to further N-nucleophiles (maleimide and glutarimide anions) and prototype C, P, O, and S-nucleophiles (namely, [(Me-CO)_2_CH]^−^, [MeO]^−^, [MeS]^−^, [Me-CO-S]^−^, [Me_2_P]^−^, and [(Me-CO)_2_P]^−^ anions) along with the F^–^, Cl^–^, and Br^–^ halide anions.^54–56^ According to these results, the above-discussed AE mechanism is also applicable for these additional nucleophiles (for details, see Figures S9–S14). Moreover, we investigated the relationship between ε_HOMO_ and ΔG_CT_. As illustrated in Figure 4 (red, unfilled dots), these additional points are in good agreement with the blue trendline (R^2^ = 0.91, if every data point is considered, see Figure S15), outlining a trend across a wide range of nucleophilic centers that the thermodynamical parameters of the reactions depend linearly on the ε_HOMO_ of the nucleophile. While some of the additional data points do not strictly follow the trendline of the N-centered anions, the linear tendency for all data points is visible.

## Conclusions

In conclusion, we have elucidated the mechanism of the nucleophilic substitution reactions between different nitrogen-containing anions and elemental iodine. Our calculations have revealed that all these reactions follow an addition–elimination-type pathway through a charge-transfer complex. These results contradict the mechanism previously reported for the same reactions, which were assumed to proceed through a central transition state. In vacuum, the overall substitution reactions are generally hampered thermodynamically because of the overstabilization of the [R_2_N–I_2_]^−^ charge-transfer complexes formed from the [R_2_N]^−^ anions and I_2_. We have demonstrated that polar solvents, such as water, are essential to achieve the necessary thermodynamic driving force, as the medium significantly affects the relative stability of the formed transient adducts. In addition, we obtained good linear correlations between the reaction energies of the substitution reactions and specific properties of the attacking anions (HOMO energy and proton affinity). As demonstrated, a similar trend is also applicable for the attack of other nucleophiles, beyond the initial set of amide anions.

Altogether, our results extend the concept of addition–elimination type substitutions to iodine centers, providing a framework to reinterpret the feasibility of such reactions prior to experiments and to plan future substitution reactions at other halogen centers. The presented mechanistic insights may assist the design of hydrolysis-resistant iodo-amines or further iodine compounds suitable for electrochemical systems utilizing the four-electron 2I^–^/I_2_/2I^+^ redox process, which are relevant for the development of high-energy-density iodine batteries.

## Computational Methods

All DFT and *ab initio* calculations as well as the Mayer population analysis were carried out using the ORCA 6.1.0 suite of programs.^57,58^ The figures presented in the main text and in the Supporting Information were rendered using CYLview20.^59^

The geometry optimizations and harmonic frequency analyses were carried out applying different DFT-based (ωB97X-D4, ωB97M-D4, M06–2X and ωB2PLYP) methods, combined with the def2-TZVPPD basis set. To obtain high-level relative energies, subsequent single-point calculations were performed at the state-of-the-art DLPNO–CCSD­(T)/def2-TZVPPD. For all calculations in this study, the RIJCOSX approximation was utilized, for this we applied the def2/J as well as the def2-TZVPPD/C auxiliary basis sets (for the DLPNO–CCSD­(T) calculations). The solvation effects were accounted for by the SMD and the CPCM implicit solvent models for water, acetonitrile, and dimethyl sulfoxide. The HOMO energies were obtained at the ωB97X-D3­(BJ)/def2-TZVPPD//B3LYP-D3­(BJ)/def2-TZVPPD level of theory.

In cases where it was reasonable to explore the conformational space, we used the CREST program^60^ in combination with the GFN2-XTB method.^61^ The obtained conformers were then fully optimized at the above DFT level of theory, and that with the lowest electronic energy was used in further calculations in each case.

Besides traditional transition state searches, we attempted to locate transition states using an alternative method, the Nudged Elastic Band (NEB) method,^62,63^ as implemented in ORCA 6.1.0. This study was carried out at the B3LYP-D3­(BJ)/def2-TZVPPD level of theory for the three most relevant anions, **4** ^**–**^, **5** ^**–**^ and **6** ^**–**^, included also in the original paper by Fan et al.^46^

## Supplementary Material



## Data Availability

The optimized geometries and corresponding electronic energies are available free of charge at 10.5281/zenodo.18911725.

## Notes

Detailed computational methods section and additional results, supplementary data, and the results of the computations presented in the paper (PDF)

## References

[1] Shaik, S. S. ; Schlegel, H. B. ; Wolfe, S. The S_N_2 Reaction. In Theoretical Aspects of Phisical Organic Chemsitry; Wiley: New York, 1992.

[2] March, J. Advanced Organic Chemistry, 4th ed.; Wiley: New York, 1992.

[3] Clayden, J. ; Greeves, N. ; Warren, S. ; Wothers, P. Organic Chemistry, 2nd ed.; Oxford University Press: Oxford, 2012.

[4] Hamlin T. A., Swart M., Bickelhaupt F. M. (2018). Nucleophilic
Substitution (S_N_2): Dependence on Nucleophile, Leaving
Group, Central Atom,
Substituents, and Solvent. ChemPhysChem.

[5] Walden P. (1896). Ueber Die
Gegenseitige Umwandlung Optischer Antipoden. Ber. Dtsch. Chem. Ges..

[6] Lovinger G. J., Sak M. H., Jacobsen E. N. (2024). Catalysis
of an S_N_2 Pathway
by Geometric Preorganization. Nature.

[7] Rezayee N. M., Enemærke V. J., Linde S. T., Lamhauge J. N., Reyes-Rodriguez G. J., Jørgensen K. A., Lu C., Houk K. N. (2021). An Asymmetric S_N_2 Dynamic Kinetic Resolution. J. Am.
Chem. Soc..

[8] O’Hagan D., Schmidberger J. W. (2010). Enzymes That Catalyse S_N_2 Reaction Mechanisms. Nat. Prod. Rep..

[9] Dong C., Huang F., Deng H., Schaffrath C., Spencer J. B., O’Hagan D., Naismith J. H. (2004). Crystal Structure
and Mechanism of a Bacterial Fluorinating Enzyme. Nature.

[10] Yang H., Li Q., Wang S., Zhang R., Sheng X., Liao C. (2025). Bimolecular
Nucleophilic Substitution (S_N_2) Reaction Catalyzed by l-Threonine
Aldolase. J. Am. Chem. Soc..

[11] Ingold, C. K. Structure and Mechanism in Organic Chemistry, 2nd ed.; Cornell University Press: Ithaca, NY, 1969.

[12] Mikosch J., Trippel S., Eichhorn C., Otto R., Lourderaj U., Zhang J. X., Hase W. L., Weidemüller M., Wester R. (2008). Imaging Nucleophilic Substitution Dynamics. Science.

[13] Xie J., Otto R., Mikosch J., Zhang J., Wester R., Hase W. L. (2014). Identification of
Atomic-Level Mechanisms for Gas-Phase
X ^–^ + CH_3_Y S_N_2 Reactions by
Combined Experiments and Simulations. Acc. Chem.
Res..

[14] Szabó I., Czakó G. (2017). Dynamics and Novel Mechanisms of S_N_2 Reactions
on Ab Initio Analytical Potential Energy Surfaces. J. Phys. Chem. A.

[15] Phan T. B., Nolte C., Kobayashi S., Ofial A. R., Mayr H. (2009). Can One Predict
Changes from S_N_1 to S_N_2 Mechanisms?. J. Am. Chem. Soc..

[16] Li J., Beak P. (1992). Endocyclic Restriction
Test: Experimental Evaluation of the Transition
Structure Geometry of a Nucleophilic Substitution at Trivalent Phosphorus. J. Am. Chem. Soc..

[17] Lahiri S. D., Zhang G., Dunaway-Mariano D., Allen K. N. (2003). The Pentacovalent
Phosphorus Intermediate of a Phosphoryl Transfer Reaction. Science.

[18] van
Bochove M. A., Swart M., Bickelhaupt F. M. (2006). Nucleophilic
Substitution at Phosphorus (S_N_2@P): Disappearance and Reappearance
of Reaction Barriers. J. Am. Chem. Soc..

[19] Damrauer R., Hankin J. A. (1995). Chemistry and Thermochemistry
of Silicon-Containing
Anions in the Gas Phase. Chem. Rev..

[20] Bento A. P., Solà M., Bickelhaupt F. M. (2005). *Ab Initio* and DFT
Benchmark Study for Nucleophilic Substitution at Carbon (S_N_2@C) and Silicon (S_N_2@Si). J. Comput.
Chem..

[21] Pappas J. A. (1977). Theoretical
Studies of the Reactions of the Sulfur-Sulfur Bond. 1. General Heterolytic
Mechanisms. J. Am. Chem. Soc..

[22] Bachrach S. M., Gailbreath B. D. (2001). Theoritical Study of Nucleophilic Substitution at Two-Coordinate
Sulfur. J. Org. Chem..

[23] Bachrach S. M., Mulhearn D. C. (1996). Nucleophilic Substitution
at Sulfur: S_N_2
or Addition–Elimination?. J. Phys. Chem..

[24] Watt G.W., Foerster D.R. (1960). Reactions of Iodine
in Liquid Ammonia. J. Inorg. Nucl. Chem..

[25] McAlpine R. K. (1952). The Reaction
of Dilute Iodine and Ammonia Solutions. J. Am.
Chem. Soc..

[26] Audrieth L. F., Birr E. J. (1933). Anomalous Electrolytes. I. The Electrical Conductivity
of Solutions of Iodine and Cyanogen Iodide in Pyridine. J. Am. Chem. Soc..

[27] Tassaing T., Besnard M. (1997). Ionization Reaction
in Iodine/Pyridine Solutions: What
Can We Learn from Conductivity Measurements, Far-Infrared Spectroscopy,
and Raman Scattering?. J. Phys. Chem. A.

[28] Reid C., Mulliken R. S. (1954). Molecular Compounds
and Their Spectra. IV. The Pyridine-Iodine
System. J. Am. Chem. Soc..

[29] Cotton F. A., Kibala P. A. (1987). Reactions of Iodine
with Triphenylphosphine and Triphenylarsine. J. Am. Chem. Soc..

[30] Yu S., Ward J. S. (2022). Ligand Exchange
among Iodine­(l) Complexes. Dalton Trans..

[31] Wang C., Danovich D., Chen H., Shaik S. (2019). Oriented External Electric
Fields: Tweezers and Catalysts for Reactivity in Halogen-Bond Complexes. J. Am. Chem. Soc..

[32] Zhu C., Pham L. N., Yuan X., Ouyang H., Coote M. L., Zhang X. (2023). High Electric Fields
on Water Microdroplets Catalyze Spontaneous
and Fast Reactions in Halogen-Bond Complexes. J. Am. Chem. Soc..

[33] Zhang X., Ren J., Min Tan S., Tan D., Lee R., Tan C.-H. (2019). An Enantioconvergent
Halogenophilic Nucleophilic Substitution (S_N_2X) Reaction. Science.

[34] Kuo L. H., Ban X., He J. H., Pham D. N. P., Kee C. W., Tan C. H. (2024). A Quantitative
Study of the Halogenophilic Nucleophilic Substitution (S_N_2X) Reaction and Chalcogenophilic Nucleophilic Substitution (S_N_2Ch) Reaction. J. Am. Chem. Soc..

[35] Lee R., Eric Chao C. B., Ban X., Tan S. M., Yu H., Hyland C. J. T., Tan C.-H. (2022). Direct
S_N_2 or S_N_2X Manifold—Mechanistic Study
of Ion-Pair-Catalyzed Carbon­(sp^3^)–Carbon­(sp^3^) Bond Formation. J. Org. Chem..

[36] Montanari S., Paradisi C., Scorrano G. (1993). Thiol Anions in Nucleophilic Aromatic
Substitution Reactions with Activated Aryl Halides. Attack on Carbon
vs Attack on Halogen. J. Org. Chem..

[37] Sakakura A., Ukai A., Ishihara K. (2007). Enantioselective Halocyclization
of Polyprenoids Induced by Nucleophilic Phosphoramidites. Nature.

[38] Horibe T., Tsuji Y., Ishihara K. (2018). Thiourea–I_2_ as
Lewis Base–Lewis Acid Cooperative Catalysts for Iodochlorination
of Alkene with In Situ-Generated I–Cl. ACS Catal..

[39] Mondal, H. Halogen and Chalcogen Activation by Nucleophilic Catalysis. Chem. Eur. J. 2024, 30, e202402261 10.1002/chem.202402261. 39039960

[40] Zefirov N. S., Makhon’kov D. I. (1982). X-Philic
Reactions. Chem. Rev..

[41] Zou Y., Liu T., Du Q., Li Y., Yi H., Zhou X., Li Z., Gao L., Zhang L., Liang X. (2021). A Four-Electron Zn-I_2_ Aqueous
Battery Enabled by Reversible I^–^/I_2_/I^+^ Conversion. Nat.
Commun..

[42] Li W., Xu H., Zhang H., Wei F., Zhang T., Wu Y., Huang L., Fu J., Jing C., Cheng J., Liu S. (2023). Designing Ternary Hydrated Eutectic Electrolyte Capable of Four-Electron
Conversion for Advanced Zn–I_2_ Full Batteries. Energy Environ. Sci..

[43] Li X., Xu W., Zhi C. (2024). Halogen-Powered
Static Conversion Chemistry. Nat. Rev. Chem..

[44] Zhang S.-J., Hao J., Wu H., Kao C.-C., Chen Q., Ye C., Qiao S.-Z. (2024). Toward High-Energy-Density Aqueous Zinc–Iodine
Batteries: Multielectron Pathways. ACS Nano.

[45] Yu, H. ; Wang, Z. ; Zheng, R. ; Yan, L. ; Zhang, L. ; Shu, J. Toward Sustainable Metal-Iodine Batteries: Materials, Electrochemistry and Design Strategies. Angew. Chem., Int. Ed. 2023, 62, e202308397 10.1002/anie.202308397. 37458970

[46] Yi Z., Xu H., Yang J.-L., Li J., Xiao T., Chen H., Jiang C., Li H., Lee S. W., Fan H. J. (2025). Deciphering
S_N_2-Type Nucleophilic Substitution via Halogen-Free Intermediates
for High-Energy Zinc–Iodine Batteries. J. Am. Chem. Soc..

[47] Yi Z., Cai D.-Q., Fan H. J. (2026). Rethinking
the Four-Electron Iodine
Redox Mechanism in Zn–I_2_ Batteries. ACS Energy Lett..

[48] Cremer D., Kraka E. (1984). Chemical Bonds without
Bonding Electron Density — Does the
Difference Electron-Density Analysis Suffice for a Description of
the Chemical Bond?. Angew. Chem., Int. Ed..

[49] Pimentel G. C. (1951). The Bonding
of Trihalide and Bifluoride Ions by the Molecular Orbital Method. J. Chem. Phys..

[50] Desiraju G. R., Ho P. S., Kloo L., Legon A. C., Marquardt R., Metrangolo P., Politzer P., Resnati G., Rissanen K. (2013). Definition
of the Halogen Bond (IUPAC Recommendations 2013). Pure Appl. Chem..

[51] Cavallo G., Metrangolo P., Milani R., Pilati T., Priimagi A., Resnati G., Terraneo G. (2016). The Halogen Bond. Chem. Rev..

[52] Mikołajczyk, M. Nucleophilic Substitution at Phosphorus Centres – Old and Recent Studies and a Final Solution of Mechanistic and Related Stereochemical Problems. Chem. Eur. J. 2024, 30, e202302974 10.1002/chem.202302974. 38116824

[53] Bento A. P., Bickelhaupt F. M. (2007). Nucleophilic Substitution at Silicon (S_N_2@Si) via a Central Reaction Barrier. J. Org.
Chem..

[54] Tanner D. D., Gidley G. C., Das N., Rowe J. E., Potter A. (1984). On the Structure
of *Tert*-Butyl Hypoiodite. J.
Am. Chem. Soc..

[55] Kummari A., Pappuru S., Singha Roy S., Chakraborty D. (2022). Iodine and
Alkali Metal Alkoxides: A Simple and Versatile Catalytic System for
Fully Alternating Polyester Synthesis from Phthalic Anhydride and
Epoxides. Polym. Chem..

[56] Shum L. G. S., Benson S. W. (1983). Thermochemistry
and Kinetics of the Reaction of Methyl
Mercaptan with Iodine. Int. J. Chem. Kinet..

[57] Neese, F. Software Update: The ORCA Program System—Version 6.0. WIREs Comp. Mol. Sci. 2025, 15, e70019 10.1002/wcms.70019.

[58] Neese F., Wennmohs F., Becker U., Riplinger C. (2020). The ORCA Quantum
Chemistry Program Package. J. Chem. Phys..

[59] Legault, C. Y. CYLview20. Université de Sherbrooke: http://www.cylview.org 2020.

[60] Pracht, P. ; Grimme, S. ; Bannwarth, C. ; Bohle, F. ; Ehlert, S. ; Feldmann, G. ; Gorges, J. ; Müller, M. ; Neudecker, T. ; Plett, C. ; Spicher, S. ; Steinbach, P. ; Wesołowski, P. A. ; Zeller, F. CREST—A Program for the Exploration of Low-Energy Molecular Chemical Space. J. Chem. Phys. 2024, 160, 114110 10.1063/5.0197592.38511658

[61] Bannwarth C., Ehlert S., Grimme S. (2019). GFN2-XTB—An Accurate and Broadly
Parametrized Self-Consistent Tight-Binding Quantum Chemical Method
with Multipole Electrostatics and Density-Dependent Dispersion Contributions. J. Chem. Theory Comput..

[62] Mills G., Jónsson H., Schenter G. K. (1995). Reversible Work Transition State
Theory: Application to Dissociative Adsorption of Hydrogen. Surf. Sci..

[63] Henkelman G., Jónsson H. (2000). Improved Tangent
Estimate in the Nudged Elastic Band
Method for Finding Minimum Energy Paths and Saddle Points. J. Chem. Phys..

