# Supplementary material for: The Mechanism of Nucleophilic Substitution Reactions at an Iodine Center: Single-Well or Double-Well Potential Energy Surface?
Source: ACS Phys Chem Au. 2026 Jul 18;6(5):1144–50. doi: 10.1021/acsphyschemau.6c00052 (PMC13614087; doi:10.1021/acsphyschemau.6c00052)
Supplement: Supplementary file 1 [file pg6c00052_si_001.pdf]

Supporting Information

The Mechanism of Nucleophilic Substitution Reactions at an Iodine Center: Single-Well or Double-Well Potential Energy Surface?

Ádám Horváth[a] *and Zoltán Benkő**[a,b]

[a] Department of Inorganic and Analytical Chemistry, Faculty of Chemical Technology and Biotechnology, Budapest University of Technology and Economics, Műegyetem rkp. 3., H-1111 Budapest, Hungary, E-mail: benko.zoltan@vbk.bme.hu

[b] HUN-REN-BME Computation-Driven Chemistry Research Group, Műegyetem rkp. 3., H-1111 Budapest, Hungary

Table of Contents

Details of the NEB calculations.. 3

Supplementary results... 4

Details of the scan calculations.. 25

Optimized geometries (in xyz format) and corresponding electronic energies (in a.u)........ 26

Details of the NEB calculations

The NEB method requires two initial geometries. For one-end, we used the geometry of the corresponding CT complexes ([**4**-I_2]$^-$**,** [**6-**I_2]$^-$**,** [**6**-I_2]$^-$). For the other end, either the N···I or I···I bond was elongated along the N···I···I axis compared to the CT complex. Furthermore, we also constructed more perturbed configurations to explore the potential energy surface in more dimensions. This means that we modified the I···I···N angle (to 180, 150, 120 and 90 degrees) and also the I···I···N···C(O) (or I···I···N···CH_2 for **6**$^-$) dihedral angle (to 0, 45, 90, for **4**$^-$ and **6**$^-$ and also to 0, 45, 90, 135 and 180 for **5**$^-$, see Figure S16 for details). The results are available under the corresponding Zenodo record (10.5281/zenodo.18911725).

The resulting structures (highest energy images) from all these calculations were subjected to frequency calculations at the same DFT level. All the structures exhibited at least one imaginary mode with a frequency below 100*i* cm^{-1}. These frequencies were then used for transition state searches using eigenmode-following, however, these attempts remained unsuccessful. Considering the low complexity of these systems, these results strengthen the conclusions based on scan calculations in main text.

Supplementary results

Table S1 Comparison of the formation *electronic energies* for the investigated CT complexes (ΔE_{CT}: $R_2N^- + I_2 \rightarrow [R_2N\text{-}I_2]^-$) and the reaction energies for the gross reaction (ΔE_{rxn}: $R_2N^- + I_2 \rightarrow R_2N\text{-}I + I^-$) calculated using different DFT-based methods combined with the def2-TZVPP basis set. The DLPNO-CCSD(T) calculations were carried out at the geometries optimized at the B3LYP-D3BJ/def2-TZVPPD level of theory.

	Method	$\mathbf{1}^-$	$\mathbf{2}^-$	$\mathbf{3}^-$	$\mathbf{4}^-$	$\mathbf{5}^-$	$\mathbf{6}^-$
ΔE_{CT}	B3LYP-D3BJ	-83.2	-75.4	-46.7	-35.2	-50.1	-73.6
	DLPNO-CCSD(T)	-81.9	-78.1	-45.1	-33.8	-48.7	-75.1
	ωB97X-D4	-81.9	-76.5	-46.8	-33.4	-48.4	-74.3
	ωB97M-D4	-81.3	-73.4	-39.8	-24.7	-45.4	-71.1
	M06-2X	-84.0	-77.0	-43.7	-33.0	-48.2	-74.1
	ωB2PLYP	-84.7	-79.7	-45.4	-34.8	-50.9	-77.4
ΔE_{rxn}	B3LYP-D3BJ	-67.5	-60.6	-24.1	-3.9	-26.6	-59.3
	DLPNO-CCSD(T)	-69.7	-66.3	-25.1	-6.9	-28.6	-63.5
	ωB97X-D4	-65.6	-60.4	-24.1	-2.2	-24.2	-58.5
	ωB97M-D4	-70.1	-62.3	-22.0	-12.6	-26.7	-60.2
	M06-2X	-69.4	-63.0	-22.9	-3.9	-26.2	-60.7
	ωB2PLYP	-71.6	-66.9	-26.1	-7.8	-30.5	-65.1

Table S2 Comparison of the I···I and N···I interatomic distances in the CT [**1**-I_2]$^-$, [**2**-I_2]$^-$, [**3**-I_2]$^-$, [**4**-I_2]$^-$, [**5**-I_2]$^-$, [**6**-I_2]$^-$ complexes optimized with different DFT-based methods combined with the def2-TZVPPD basis set.

**CT complex**	**Method**	**I···I**	**N···I**
[**1**-I_2]$^-$	B3LYP-D3BJ	3.24	2.20
	ωB97X-D4	3.16	2.13
	ωB97M-D4	3.30	2.13
	M06-2X	3.24	2.14
	ωB2PLYP	3.24	2.10
[**2**-I_2]$^-$	B3LYP-D3BJ	3.23	2.19
	ωB97X-D4	3.15	2.12
	ωB97M-D4	3.28	2.12
	M06-2X	3.22	2.13
	ωB2PLYP	3.23	2.10
[**3**-I_2]$^-$	B3LYP-D3BJ	3.08	2.27
	ωB97X-D4	3.03	2.17
	ωB97M-D4	3.11	2.19
	M06-2X	3.05	2.21
	ωB2PLYP	3.07	2.18
[**4**-I_2]$^-$	B3LYP-D3BJ	2.97	2.30
	ωB97X-D4	2.90	2.23
	ωB97M-D4	2.95	2.25
	M06-2X	2.93	2.24
	ωB2PLYP	2.94	2.21
[**5**-I_2]$^-$	B3LYP-D3BJ	3.08	2.20
	ωB97X-D4	3.01	2.14
	ωB97M-D4	3.10	2.14
	M06-2X	3.05	2.15
	ωB2PLYP	3.07	2.12

$[\mathbf{6}\text{-}I_2]^-$	B3LYP-D3BJ	3.24	2.17
	ωB97X-D4	3.16	2.10
	ωB97M-D4	3.29	2.10
	M06-2X	3.24	2.12
	ωB2PLYP	3.23	2.09

Table S3 Comparison of SMD and CPCM solvent models with water, acetonitrile (ACN) and dimethyl sulphoxide (DMSO) solvent for $\mathbf{4}^-$, $\mathbf{5}^-$ and $\mathbf{6}^-$ anions at the B3LYP-D3BJ/def2-TZVPPD level of theory.

		$\mathbf{4}^-$			$\mathbf{5}^-$			$\mathbf{6}^-$		
		ΔE_{CT}	ΔE_{rxn}	ΔE_{diss}	ΔE_{CT}	ΔE_{rxn}	ΔE_{diss}	ΔE_{CT}	ΔE_{rxn}	ΔE_{diss}
SMD	water	-14.5	-5.2	+9.3	-25.5	-19.7	+5.8	-55.0	-52.3	+2.7
	ACN	-22.1	-13.4	+8.7	-34.6	-28.8	+5.8	-59.3	-56.3	+3.0
	DMSO	-23.2	-14.6	+8.6	-35.7	-30.1	+5.6	-59.7	-56.6	+3.1
CPCM	water	-22.4	-10.7	+11.7	-35.2	-27.3	+7.9	-62.8	-58.8	+4.0
	ACN	-22.6	-10.6	+12.0	-35.4	-27.3	+8.1	-63.0	-58.9	+4.1
	DMSO	-22.5	-10.6	+11.9	-35.3	-27.3	+8.0	-62.9	-58.2	+4.7

Table S4 Comparison of SMD and CPCM solvent models with water, acetonitrile (ACN) and dimethyl sulphoxide (DMSO) solvent for **4**⁻, **5**⁻ and **6**⁻ anions at the B3LYP-D3BJ/def2-TZVPPD level of theory.

		$\mathbf{4^-}$			$\mathbf{5^-}$			$\mathbf{6^-}$		
		ΔG_{CT}	ΔG_{rxn}	ΔG_{diss}	ΔG_{CT}	ΔG_{rxn}	ΔG_{diss}	ΔG_{CT}	ΔG_{rxn}	ΔG_{diss}
SMD	water	−3.1	−1.5	+1.6	−13.9	−15.7	-1.8	−41.9	−46.7	−4.8
	ACN	−10.6	−9.6	+1.0	−22.6	−24.5	−1.9	−46.1	−50.6	−4.5
	DMSO	−11.7	−10.7	+1.0	−23.8	−25.7	−1.9	−46.5	−50.9	−4.4
CPCM	water	−10.9	−6.9	+4.0	−23.2	−23.0	+0.2	−49.5	−53.0	−3.5
	ACN	−11.1	−6.8	+4.3	−23.4	−23.0	+0.4	−49.6	−53.0	−3.4
	DMSO	−11.0	−6.8	+4.2	−23.3	−23.0	+0.3	−49.5	−53.0	−3.5

Table S5 Comparison of the I···I and N···I interatomic distances in the CT complexes [**4**-I_2]⁻, [**5**-I_2]⁻, [**6**-I_2]⁻ applying SMD or CPCM solvent models with water, acetonitrile (ACN) and dimethyl sulphoxide (DMSO) as solvent at the B3LYP-D3BJ/def2-TZVPPD level of theory.

		**4**		**5**		**6**	
		**I···I**	**N···I**	**I···I**	**N···I**	**I···I**	**N···I**
SMD	water	3.06	2.20	3.26	2.10	3.58	2.11
	ACN	3.13	2.16	3.30	2.10	3.56	2.11
	DMSO	3.14	2.16	3.30	2.09	3.56	2.10
PCM	water	3.08	2.19	3.25	2.11	3.53	2.11
	ACN	3.08	2.19	3.25	2.11	3.53	2.11
	DMSO	3.08	2.19	3.25	2.11	3.53	2.11

Table S6 Formation electronic energies of $R_2N^{\cdot}$ radical + $[I_2]^{\cdot-}$ radical anion at the B3LYP-D3BJ/def2-TZVPPD level of theory.

Anion	ΔE
**1**⁻	−50.6
**2**⁻	−54.3
**3**⁻	−6.3
**4**⁻	+28.3
**5**⁻	−7.2
**6**⁻	−53.4

Table S7 Formation *Gibbs free energies* for the investigated CT complexes (ΔG_{CT}: $R_2N^- + I_2 \rightarrow [R_2N\text{-}I_2]^-$), the reaction *Gibbs free energies* for the gross reaction (ΔG_{rxn}: $R_2N^- + I_2 \rightarrow R_2N\text{-}I + I^-$), and for the dissociation of the CT complexes (ΔG_{diss}: $[R_2N\text{-}I_2]^- \rightarrow R_2N\text{-}I + I^-$). The values were obtained at the **B3LYP-D3BJ/def2-TZVPPD** level of theory both in vacuum and using SMD implicit solvation model and are given in kcal mol^{-1}.

Anion	ΔG_{CT}		ΔG_{rxn}		ΔG_{diss}	
	vacuum	solvent	vacuum	solvent	vacuum	solvent
**1**⁻	−72.4	−39.9	−63.2	−43.6	+9.2	−3.7
**2**⁻	−61.9	−41.7	−53.8	−46.1	+8.1	−4.4
**3**⁻	−33.5	−27.0	−18.3	−30.7	+15.2	−3.7
**4**⁻	−24.0	−3.1	−0.5	−1.5	+23.5	+1.7
**5**⁻	−37.7	−13.9	−21.5	−15.7	+16.2	−1.8
**6**⁻	−59.6	−41.9	−52.4	−46.7	+7.2	−4.8

Table S8 Formation *electronic energies* for the investigated CT complexes (ΔE_{CT}: $R_2N^- + I_2 \rightarrow [R_2N\text{-}I_2]^-$), the reaction energies for the gross reaction (ΔE_{rxn}: $R_2N^- + I_2 \rightarrow R_2N\text{-}I + I^-$), and for the dissociation of the CT complexes (ΔE_{diss}: $[R_2N\text{-}I_2]^- \rightarrow R_2N\text{-}I + I^-$). The values were obtained at the **B3LYP-D3BJ/def2-TZVPPD** level of theory, both in vacuum and using SMD implicit solvation model and are given in kcal mol^{-1}.

Anion	ΔE_{CT}		ΔE_{rxn}		ΔE_{diss}	
	vacuum	solvent	vacuum	solvent	vacuum	solvent
**1**$^-$	−83.2	−51.1	−67.5	−47.9	+15.8	+3.2
**2**$^-$	−75.4	−54.7	−60.6	−51.8	+14.8	+2.9
**3**$^-$	−46.7	−40.3	−24.1	−36.3	+22.6	+2.9
**4**$^-$	−35.2	−14.5	−3.9	−5.2	+31.3	+9.3
**5**$^-$	−50.1	−25.5	−26.6	−19.7	+23.5	+5.8
**6**$^-$	−73.6	−55.0	−59.3	−52.3	+14.4	+2.7

Table S9 Formation *electronic energies* for the investigated CT complexes (ΔE_{CT}: $R_2N^- + I_2 \rightarrow [R_2N\text{-}I_2]^-$), the reaction energies for the gross reaction (ΔE_{rxn}: $R_2N^- + I_2 \rightarrow R_2N\text{-}I + I^-$), and for the dissociation of the CT complexes (ΔE_{diss}: $[R_2N\text{-}I_2]^- \rightarrow R_2N\text{-}I + I^-$). The values were obtained at the **DLPNO-CCSD(T)/def2-TZVPPD**//B3LYP-D3BJ/def2-TZVPPD level of theory both in vacuum and using SMD implicit solvation model and are given in kcal mol^{-1}.

Anion	ΔE_{CT}		ΔE_{rxn}		ΔE_{diss}	
	vacuum	solvent	vacuum	solvent	vacuum	solvent
**1**$^-$	−81.9	−52.0	−69.7	−50.5	+12.1	+1.5
**2**$^-$	−78.1	−57.8	−66.3	−56.1	+11.9	+2.9
**3**$^-$	−45.1	−40.4	−25.1	−33.2	+20.0	+7.2
**4**$^-$	−33.8	−13.4	−6.9	−8.1	+26.9	+5.3
**5**$^-$	−48.7	−23.9	−28.6	−21.5	+20.1	+2.4
**6**$^-$	−75.1	−57.7	−63.5	−55.9	+11.7	−1.8

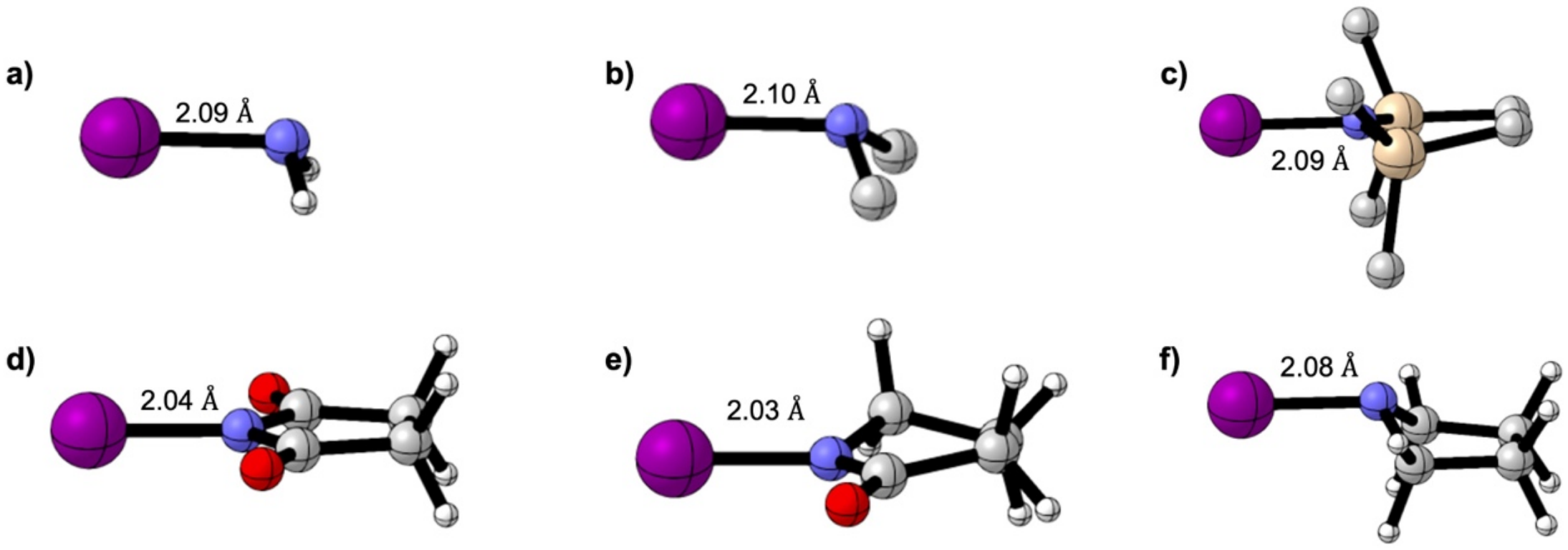

Figure S1 Gas phase structure of the R_2N-I species a)-f) for anions **1**$^-$-**6**$^-$.

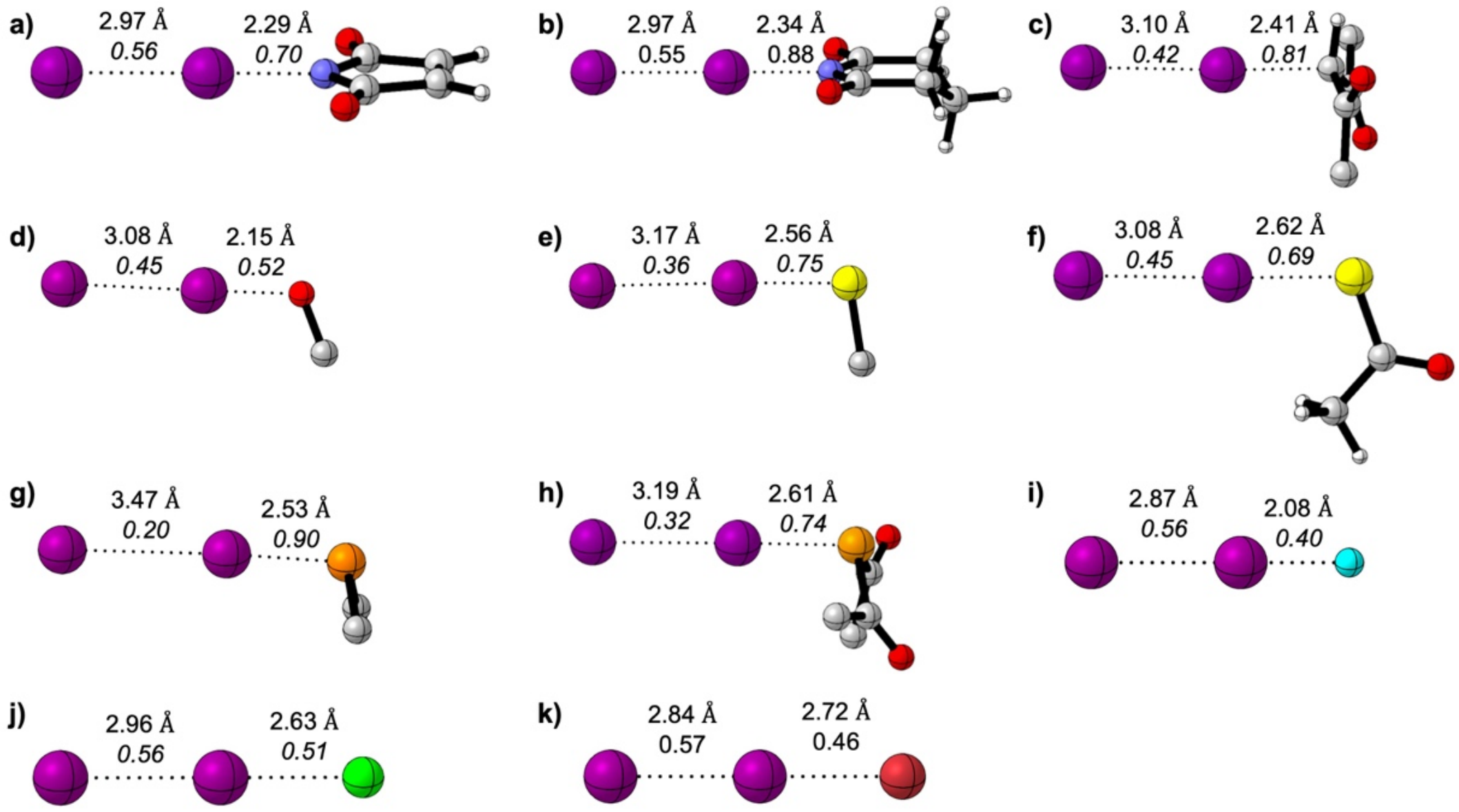

Figure S2 Gas phase structure of the $[Nu\text{-}I_2]^-$ CT complexes in the cases of the further nucleophiles Nu^-: a) maleimide, b) glutarimide, c) $[(Me\text{-}CO)_2HC]^-$, d) $[MeO]^-$, e) $[MeS]^-$, f) $[(Me\text{-}CO)S]^-$, g) $[Me_2P]^-$, h) $[(Me\text{-}CO)_2P]^-$, i) $[F]^-$, j) $[Cl]^-$, k) $[Br]^-$ anion.

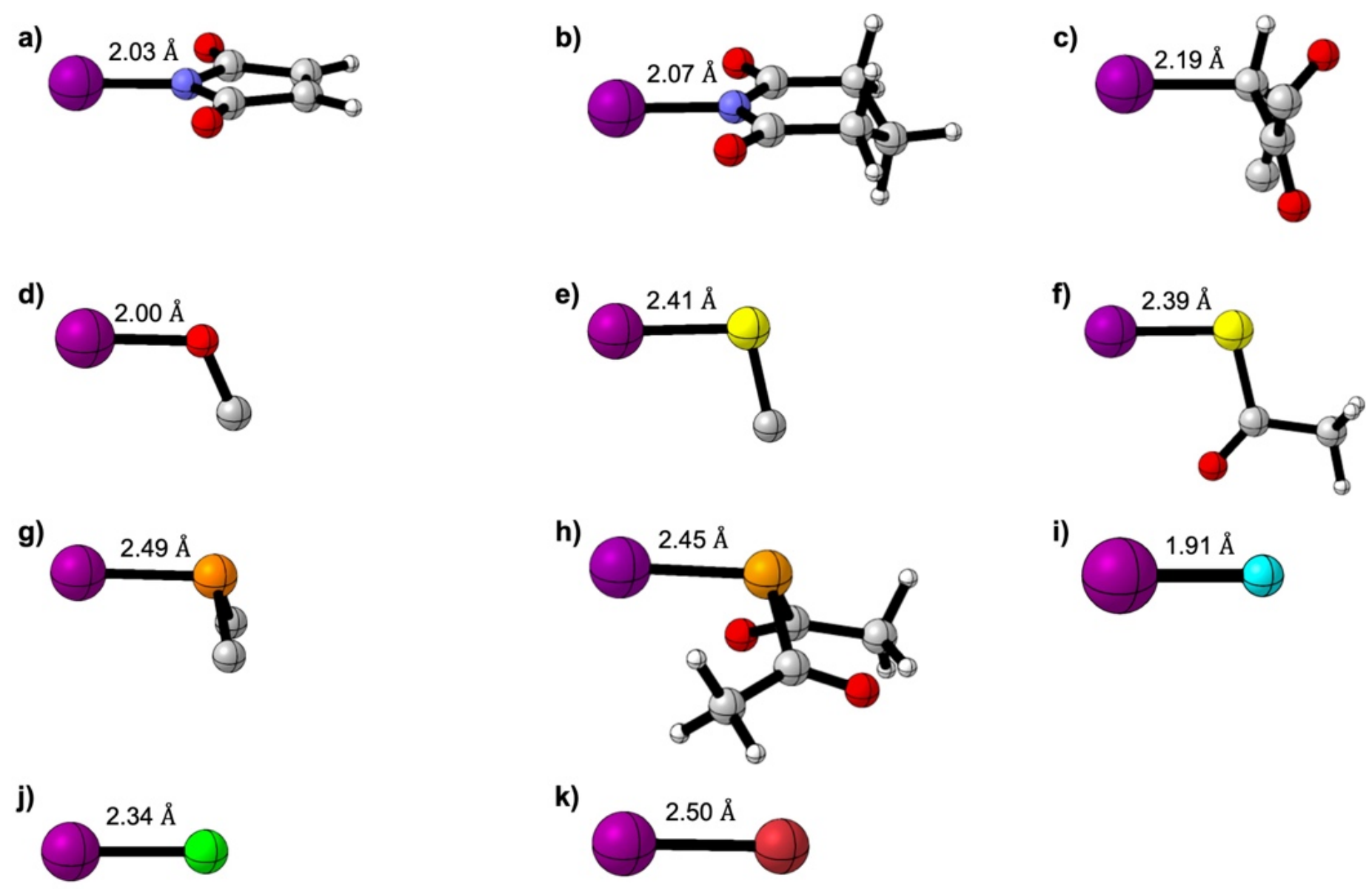

Figure S3 Gas phase structure of the Nu-I species for the further nucleophiles Nu^-: a) maleimide, b) glutarimide, c) $[(Me\text{-}CO)_2HC]^-$, d) $[MeO]^-$, e) $[MeS]^-$, f) $[(Me\text{-}CO)S]^-$, g) $[Me_2P]^-$, h) $[(Me\text{-}CO)_2P]^-$, i) $[F]^-$, j) $[Cl]^-$, k) $[Br]^-$ anion.

Table S10 Results of the Atoms-In-Molecule (AIM) analyses for the N···I and N···I interactions of the CT adducts [**1**-I_2]$^-$-[**6**-I_2]$^-$: electron density (ρ, in a.u.), its Laplacian ($\nabla^2\rho$, in a.u.) and the total energy density (H, in a.u.) at the bond critical points. The wavefunctions were obtained at the B3LYP-D3BJ/def2-TZVPPD level of theory.

CT adduct	N···I			I···I		
	ρ	$\nabla^2\rho$	H	ρ	$\nabla^2\rho$	H
**[1-**I_2**]**$^-$	0.0978	0.0673	−0.037	0.0275	0.0408	−0.0016
**[2-**I_2**]**$^-$	0.101	0.0668	−0.040	0.0281	0.0409	−0.0018
**[3-**I_2**]**$^-$	0.0821	0.0841	−0.026	0.0369	0.0438	−0.0042
**[4-**I_2**]**$^-$	0.0763	0.107	−0.022	0.0445	0.0403	−0.0070
**[5-**I_2**]**$^-$	0.0942	0.108	−0.035	0.0365	0.0439	−0.0042
**[6-**I_2**]**$^-$	0.104	0.0743	−0.042	0.0278	0.0408	−0.0017
$[I_3]^-$	-	-	-	0.0437	0.0427	−0.0064

Table S11 Atoms-In-Molecule analysis results for the bond critical point of the I···I and N···I interaction along the one-dimensional scan calculations for anion **4**⁻: electron density (ρ, in a.u.), its Laplacian ($\nabla^2\rho$, in a.u.), the total energy density (H, in a.u.) and the ratio of the absolute value of the potential energy density and the Lagrangian kinetic energy (∣V∣/G, in a.u.). Negative direction corresponds to the elongation of the distance.

Step	I···I				N···I			
	ρ	$\nabla^2\rho$	H	∣V∣/G	ρ	$\nabla^2\rho$	H	∣V∣/G
−5	0.0186	0.0242	−0.0003	1.05	0.0967	0.118	−0.036	1.55
−4	0.0221	0.0280	−0.0008	1.10	0.0937	0.116	−0.034	1.54
−3	0.0263	0.0321	−0.0015	1.16	0.0901	0.114	−0.032	1.53
−2	0.0312	0.0362	−0.0027	1.23	0.0861	0.112	−0.029	1.51
−1	0.0373	0.0399	−0.0044	1.31	0.0815	0.109	−0.026	1.48
CT	0.0445	0.0403	−0.0070	1.40	0.0763	0.107	−0.022	1.45
1	0.0485	0.0404	−0.0087	1.46	0.0614	0.091	−0.013	1.37
2	0.0519	0.0380	−0.0102	1.52	0.0494	0.080	−0.0076	1.28
3	0.0548	0.0355	−0.0116	1.57	0.0397	0.070	−0.0040	1.19
4	0.0571	0.0332	−0.0128	1.61	0.0320	0.060	−0.0018	1.10
5	0.0592	0.0310	−0.0139	1.64	0.0258	0.051	−0.0004	1.03

Table S12 Atoms-In-Molecule analysis results for the bond critical point of the I···I and N···I interaction along the one-dimensional scan calculations for anion **5**⁻: electron density (ρ, in a.u.), its Laplacian ($\nabla^2\rho$, in a.u.), the total energy density (H, in a.u.) and the ratio of the absolute value of the potential energy density and the Lagrangian kinetic energy (│V│/G, in a.u.). Negative direction corresponds to the elongation of the distance.

Step	I···I				N···I			
	ρ	$\nabla^2\rho$	H	│V│/G	ρ	$\nabla^2\rho$	H	│V│/G
−5	0.0189	0.0271	−0.0003	1.05	0.107	0.114	−0.044	1.61
−4	0.0215	0.0303	−0.0007	1.08	0.105	0.113	−0.043	1.60
−3	0.0246	0.0336	−0.0012	1.12	0.103	0.112	−0.041	1.60
−2	0.0280	0.0371	−0.0019	1.17	0.100	0.111	−0.039	1.59
−1	0.0320	0.0406	−0.0028	1.22	0.0973	0.109	−0.037	1.58
CT	0.0365	0.0439	−0.0042	1.28	0.0942	0.108	−0.035	1.56
1	0.0404	0.0434	−0.0054	1.33	0.0772	0.089	−0.023	1.51
2	0.0440	0.0424	−0.0068	1.39	0.0624	0.077	−0.014	1.42
3	0.0471	0.0409	−0.0080	1.44	0.0504	0.069	−0.008	1.32
4	0.0496	0.0394	−0.0090	1.48	0.0408	0.060	−0.004	1.23
5	0.0517	0.0379	−0.0100	1.51	0.0331	0.052	−0.002	1.15

Table S13 Atoms-In-Molecule analysis results for the bond critical point of the I···I and N···I interaction along the one-dimensional scan calculations for anion **6**⁻: electron density (ρ, in a.u.), its Laplacian ($\nabla^2\rho$, in a.u.), the total energy density (H, in a.u.) and the ratio of the absolute value of the potential energy density and the Lagrangian kinetic energy (∣V∣/G, in a.u.). Negative direction corresponds to the elongation of the distance.

Step	I···I				N···I			
	ρ	$\nabla^2\rho$	H	∣V∣/G	ρ	$\nabla^2\rho$	H	∣V∣/G
−5	0.0128	0.0209	0.0003	0.94	0.113	0.0699	−0.049	1.74
−4	0.0150	0.0241	0.0002	0.97	0.111	0.0706	−0.048	1.73
−3	0.0175	0.0277	−0.00007	1.01	0.110	0.0714	−0.047	1.72
−2	0.0204	0.0318	−0.0004	1.05	0.108	0.0722	−0.046	1.72
−1	0.0238	0.0362	−0.0010	1.10	0.106	0.0731	−0.044	1.71
CT	0.0278	0.0408	−0.0017	1.15	0.104	0.0743	−0.042	1.69
1	0.0308	0.0419	−0.0024	1.18	0.085	0.0603	−0.027	1.65
2	0.0334	0.0424	−0.0031	1.22	0.069	0.0558	−0.017	1.55
3	0.0355	0.0426	−0.0036	1.25	0.056	0.0520	−0.011	1.45
4	0.0371	0.0427	−0.0041	1.28	0.046	0.0471	−0.006	1.35
5	0.0384	0.0425	−0.0044	1.29	0.038	0.0414	−0.004	1.26

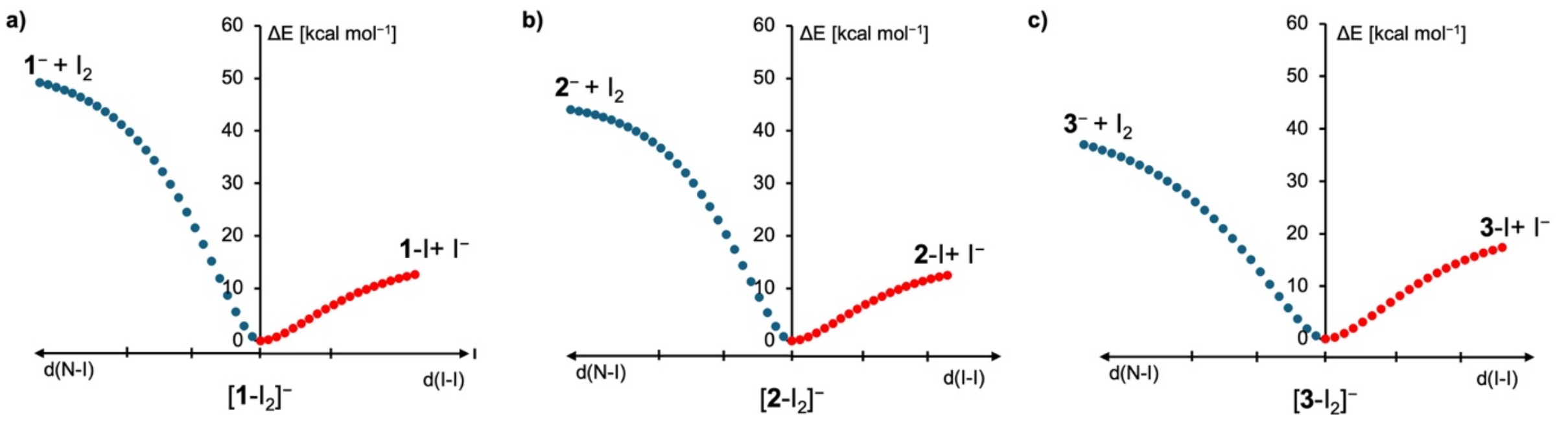

Figure S4 Relaxed 1D-scan calculations in vacuum started from the CT adducts for a) [**1**-I_2]⁻, b) [**2**-I_2]⁻ and c) [**3**-I_2]⁻.

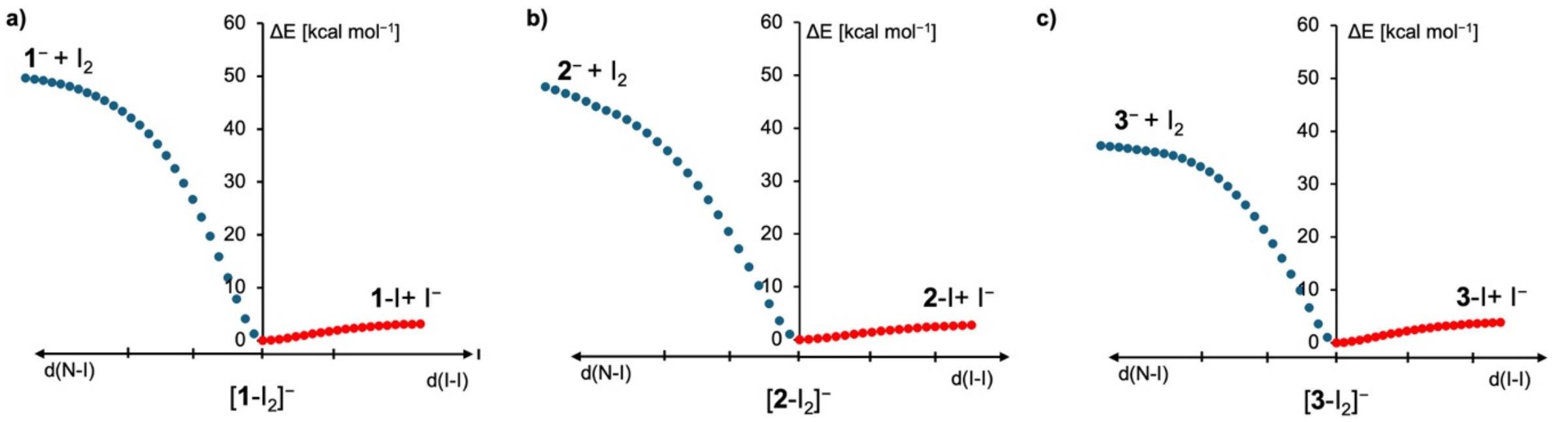

Figure S5 Relaxed 1D-scan calculations applying SMD(water) solvent model started from the CT adducts for a) [**1**-I_2]$^-$, b) [**2**-I_2]$^-$ and c) [**3**-I_2]$^-$.

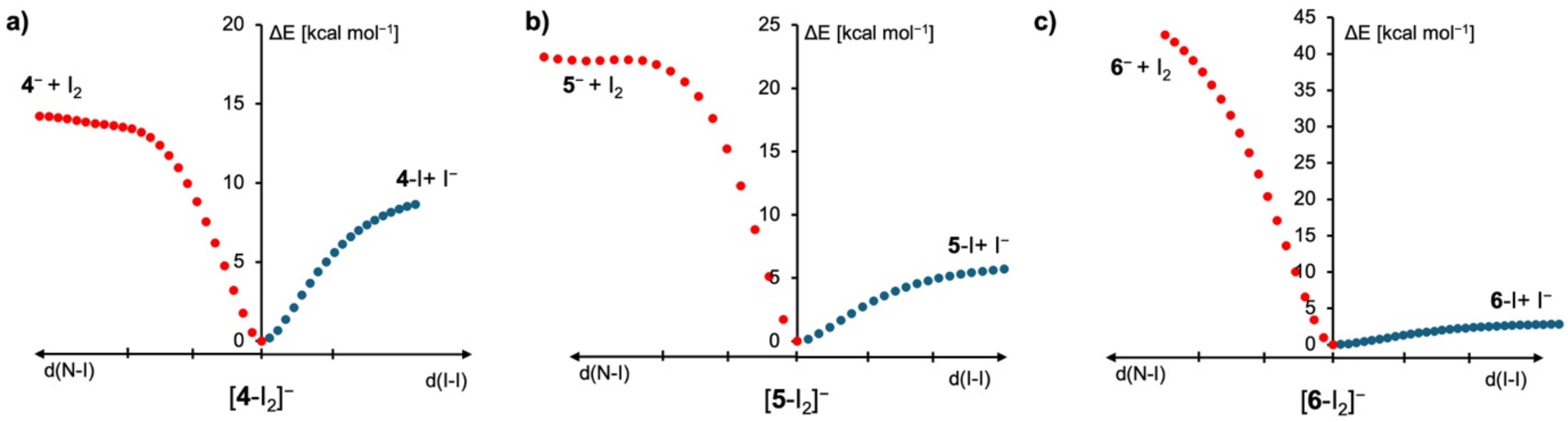

Figure S6 Relaxed 1D-scan calculations applying SMD(water) solvent model started from the CT adducts for a) [**4**-I_2]$^-$, b) [**5**-I_2]$^-$ and c) [**6**-I_2]$^-$.

Table S14 Gas phase proton affinities (PA, in kcal mol^{-1}) for anion **1**$^-$-**6**$^-$.

	PA	
**Anion**	DLPNO-CCSD(T)/def2-TZVPPD//B3LYP-D3BJ/def2-TZVPPD	B3LYP-D3BJ/def2-TZVPPD
**1**$^-$	416.5	415.3
**2**$^-$	407.1	402.1
**3**$^-$	368.5	370.1
**4**$^-$	351.3	351.0
**5**$^-$	371.8	372.1
**6**$^-$	402.3	400.3

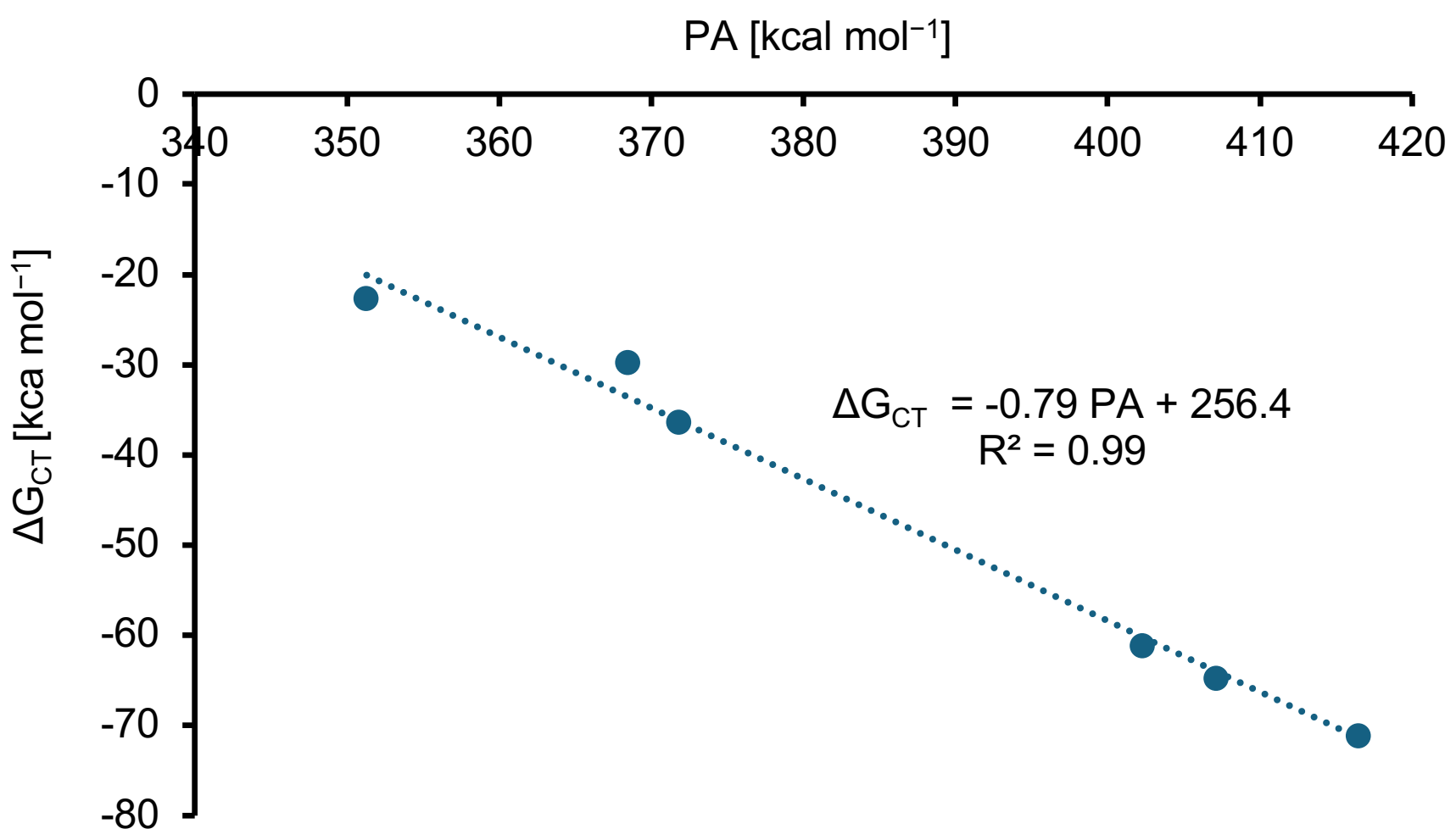

Figure S7 ΔG_{CT} (in kcal mol^{-1}) as the function of the proton affinity (PA, in kcal mol^{-1}) obtained in vacuum for anions **1**$^{-}$-**6**$^{-}$.

Table S15 HOMO energies (in eV) for all considered anions at the ωB97X-D3(BJ)/def2-TZVPPD//B3LYP-D3(BJ)/def2-TZVPPD level of theory.

Anion	HOMO energy
**1**$^{-}$	−0.59
**2**$^{-}$	−0.87
**3**$^{-}$	−3.77
**4**$^{-}$	−4.57
**5**$^{-}$	−3.01
**6**$^{-}$	−1.15
MeO^{-}	−1.72
MeS^{-}	−2.07
Me_2P^{-}	−0.79
F^{-}	−2.92
Cl^{-}	−3.49
Br^{-}	−3.43
maleimide	−4.48
glutarimide	−4.42
$[(Me\text{-}CO)S]^{-}$	−3.13
$[(Me\text{-}CO)_2P]^{-}$	−3.11
$[(Me\text{-}CO)_2HC]^{-}$	−3.30

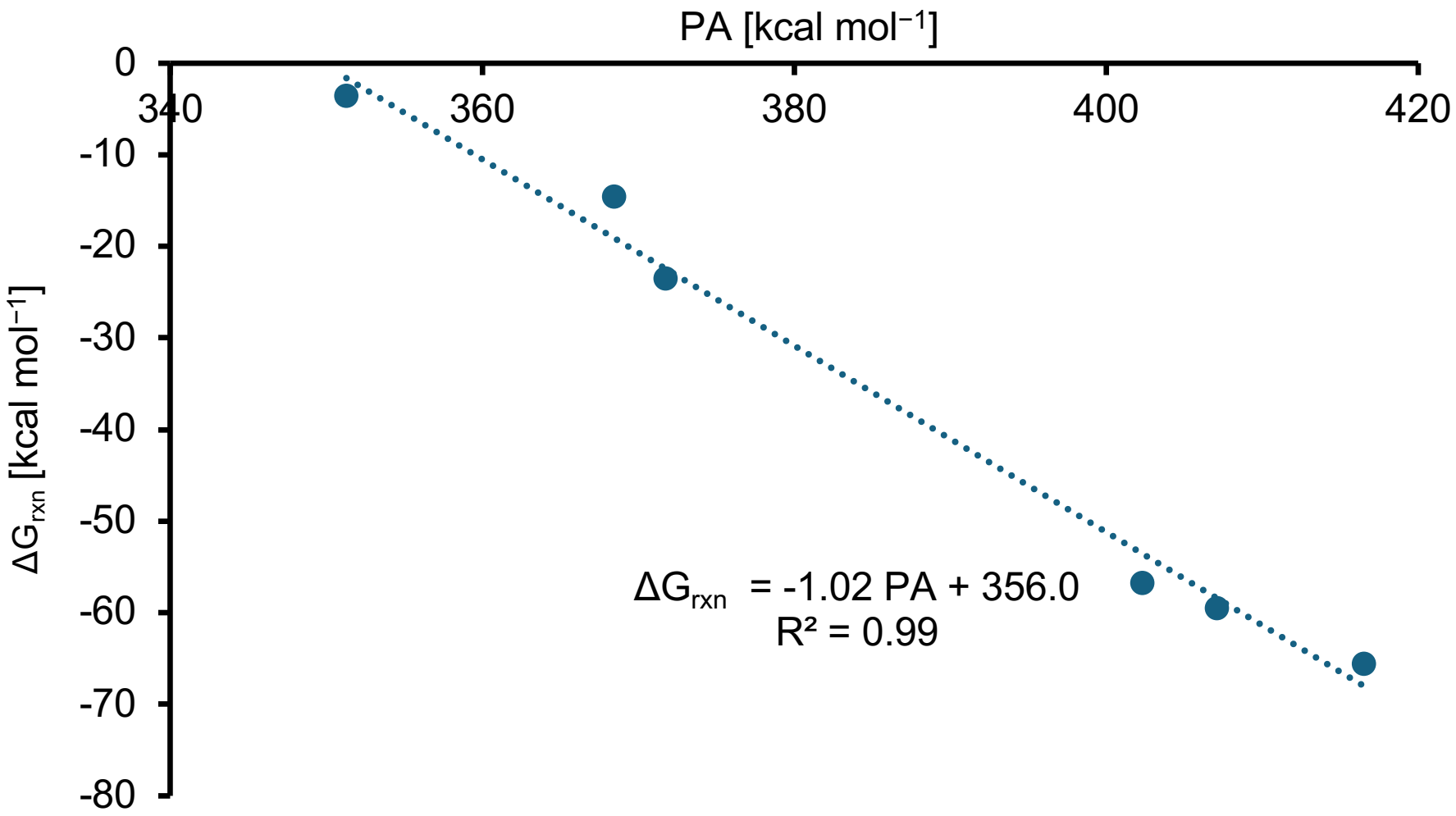

Figure S8 ΔG_{rxn} in kcal mol^{-1}) as the function of the proton affinity (PA, in kcal mol^{-1}) obtained in vacuum for anions **1**$^{-}$-**6**$^{-}$.

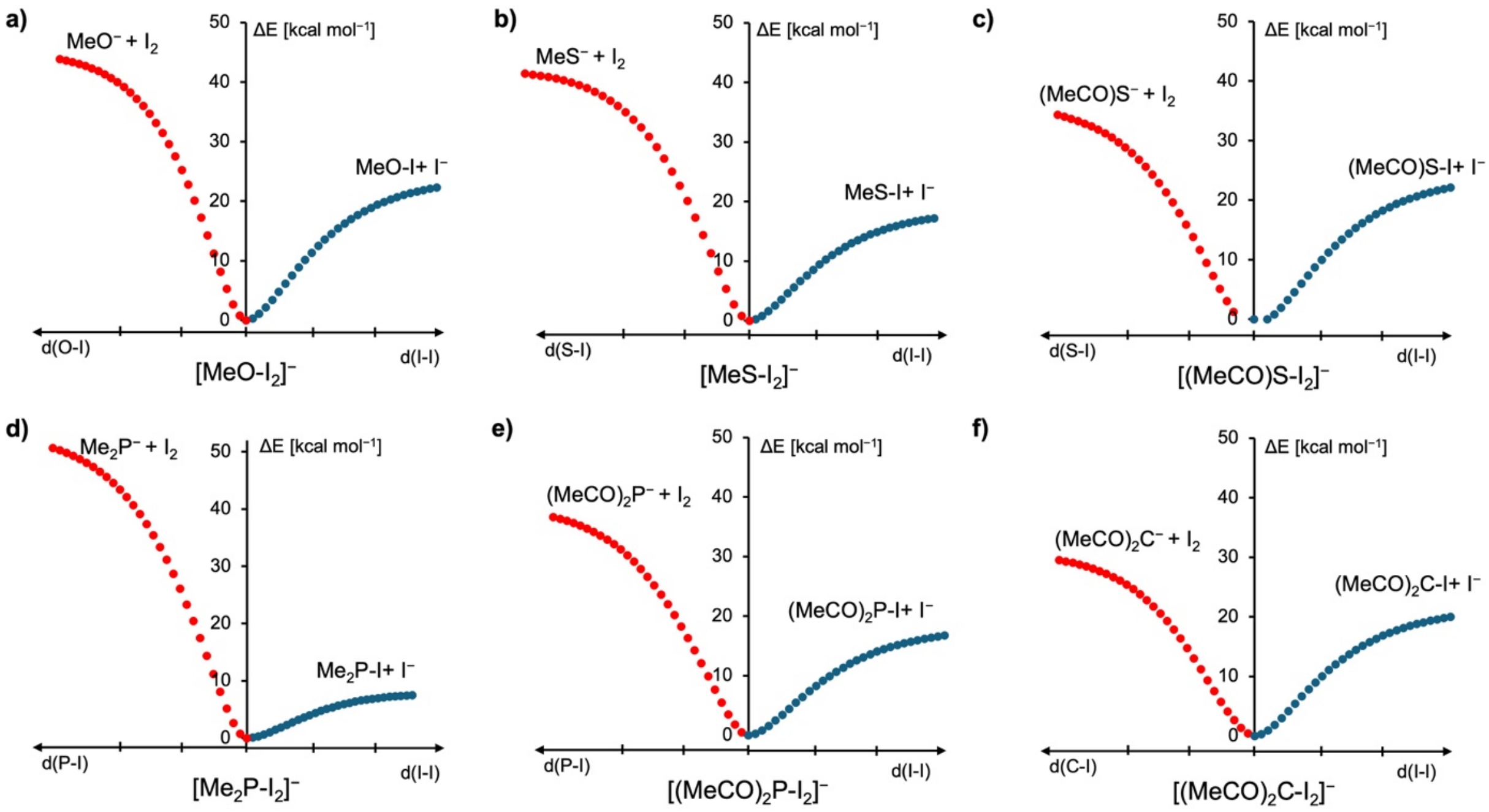

Figure S9 Relaxed 1D-scan calculations in vacuum started from the a) $[MeO\text{-}I_2]^{-}$, b) $[MeS\text{-}I_2]^{-}$, c) $[(Me\text{-}CO)S\text{-}I_2]^{-}$, d) $[Me_2P\text{-}I_2]^{-}$, e) $[(Me\text{-}CO)_2P\text{-}I_2]^{-}$, f) $[(Me\text{-}CO)_2HC\text{-}I_2]^{-}$ CT complexes.

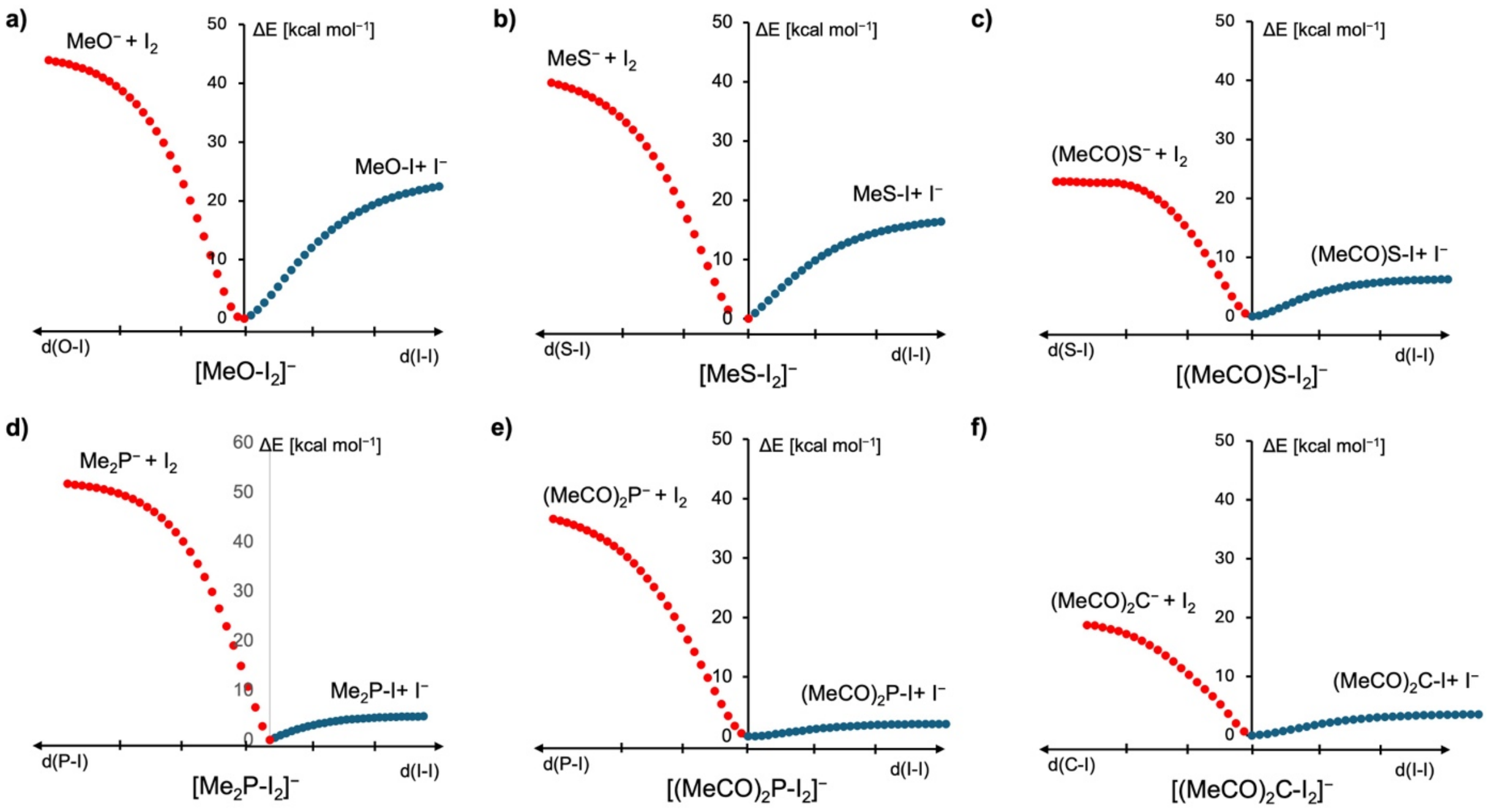

Figure S10 Relaxed 1D-scan calculations applying SMD solvent model started from the a) $[MeO\text{-}I_2]^-$, b) $[MeS\text{-}I_2]^-$, c) $[(Me\text{-}CO)S\text{-}I_2]^-$, d) $[Me_2P\text{-}I_2]^-$, e) $[(Me\text{-}CO)_2P\text{-}I_2]^-$, f) $[(Me\text{-}CO)_2HC\text{-}I_2]^-$ CT complexes.

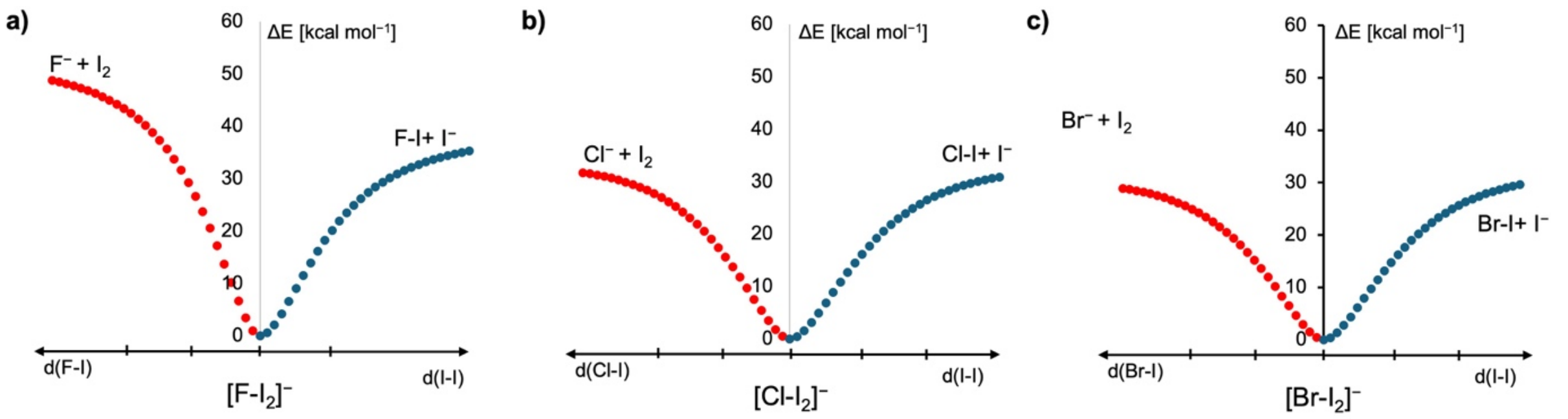

Figure S11 Relaxed 1D-scan calculations in vacuum started from the a) $[F\text{-}I_2]^-$, b) $[Cl\text{-}I_2]^-$ and c) $[Br\text{-}I_2]^-$ CT complexes.

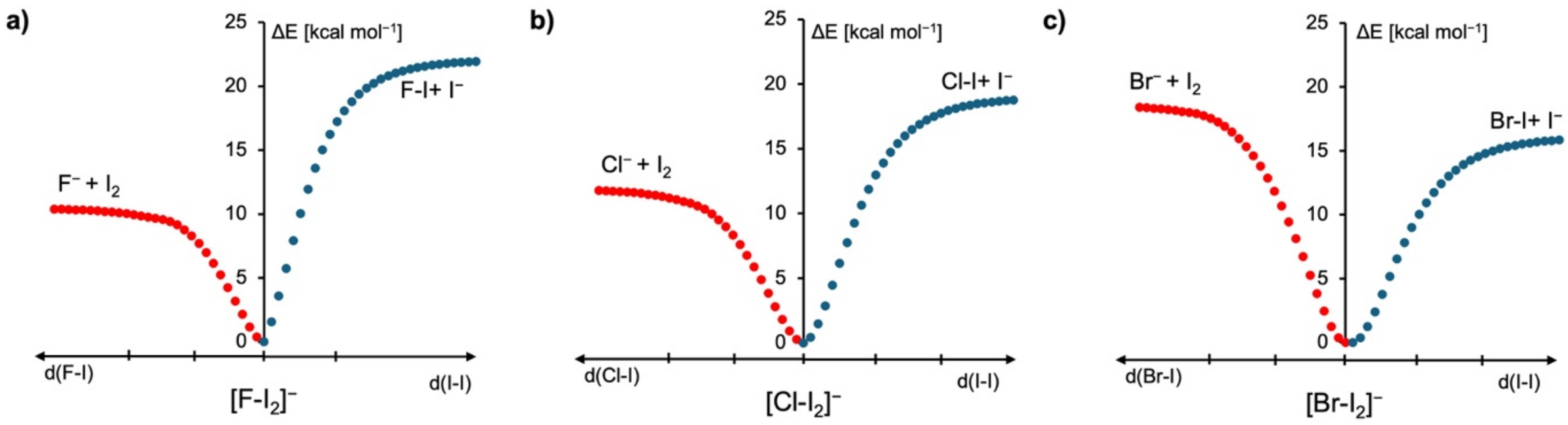

Figure S12 Relaxed 1D-scan calculations applying SMD solvent model started from the a) $[F\text{-}I_2]^-$, b) $[Cl\text{-}I_2]^-$ and c) $[Br\text{-}I_2]^-$ CT complexes.

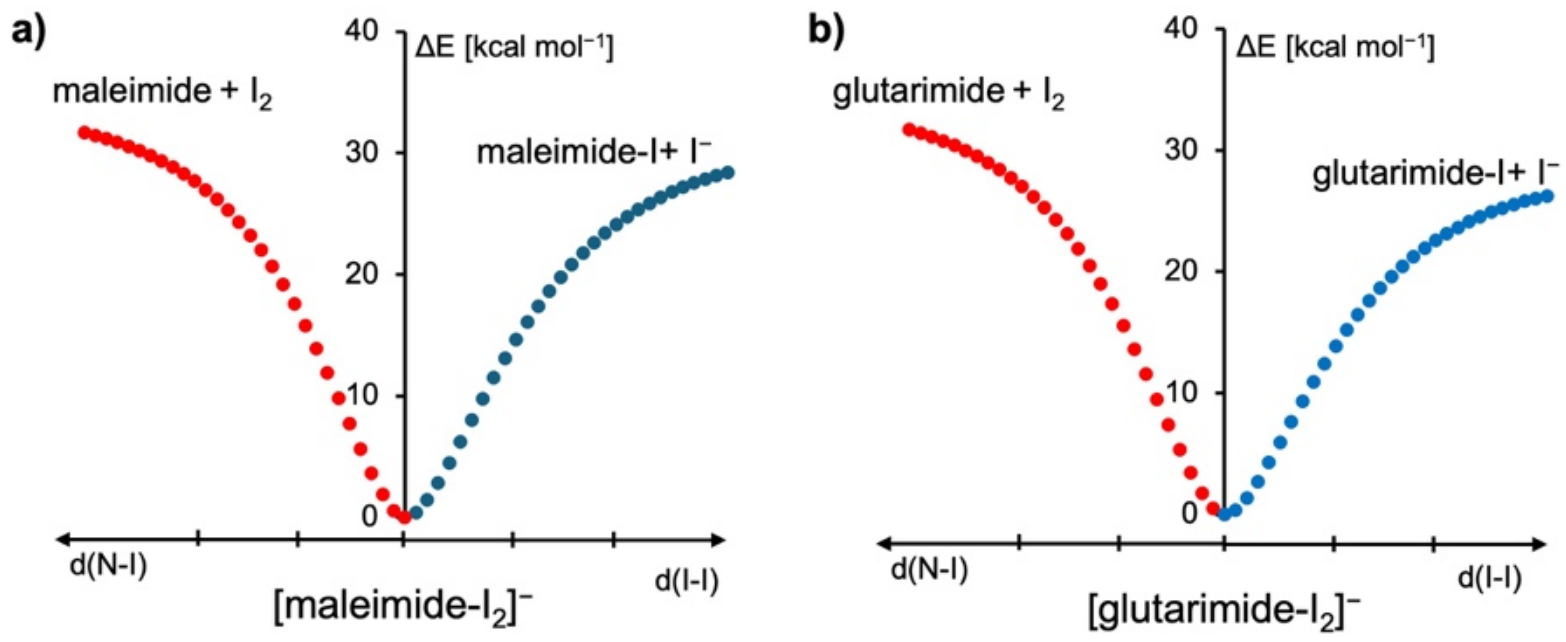

Figure S13 Relaxed 1D-scan calculations in vacuum started from the CT complex of a) maleimide and b) glutarimide anion.

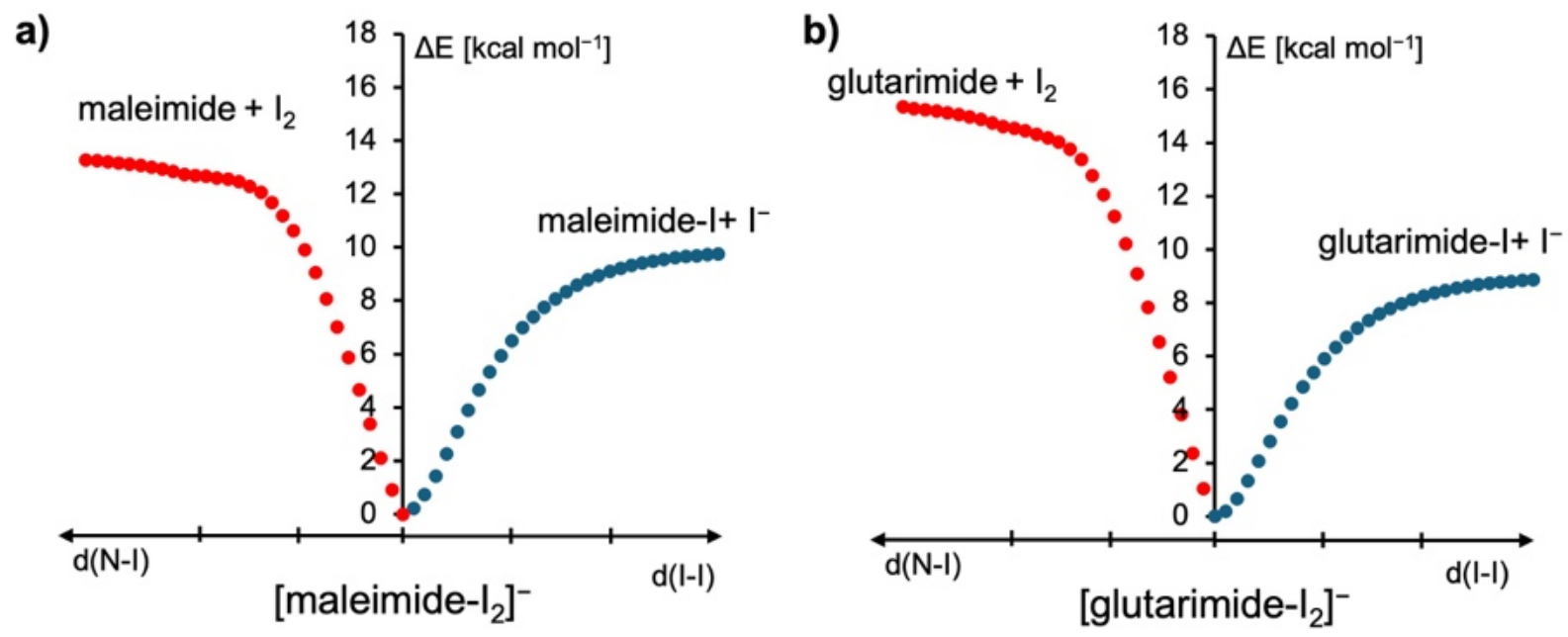

Figure S14 Relaxed 1D-scan calculations applying SMD solvent model started from the CT complex of a) maleimide and b) glutarimide anion.

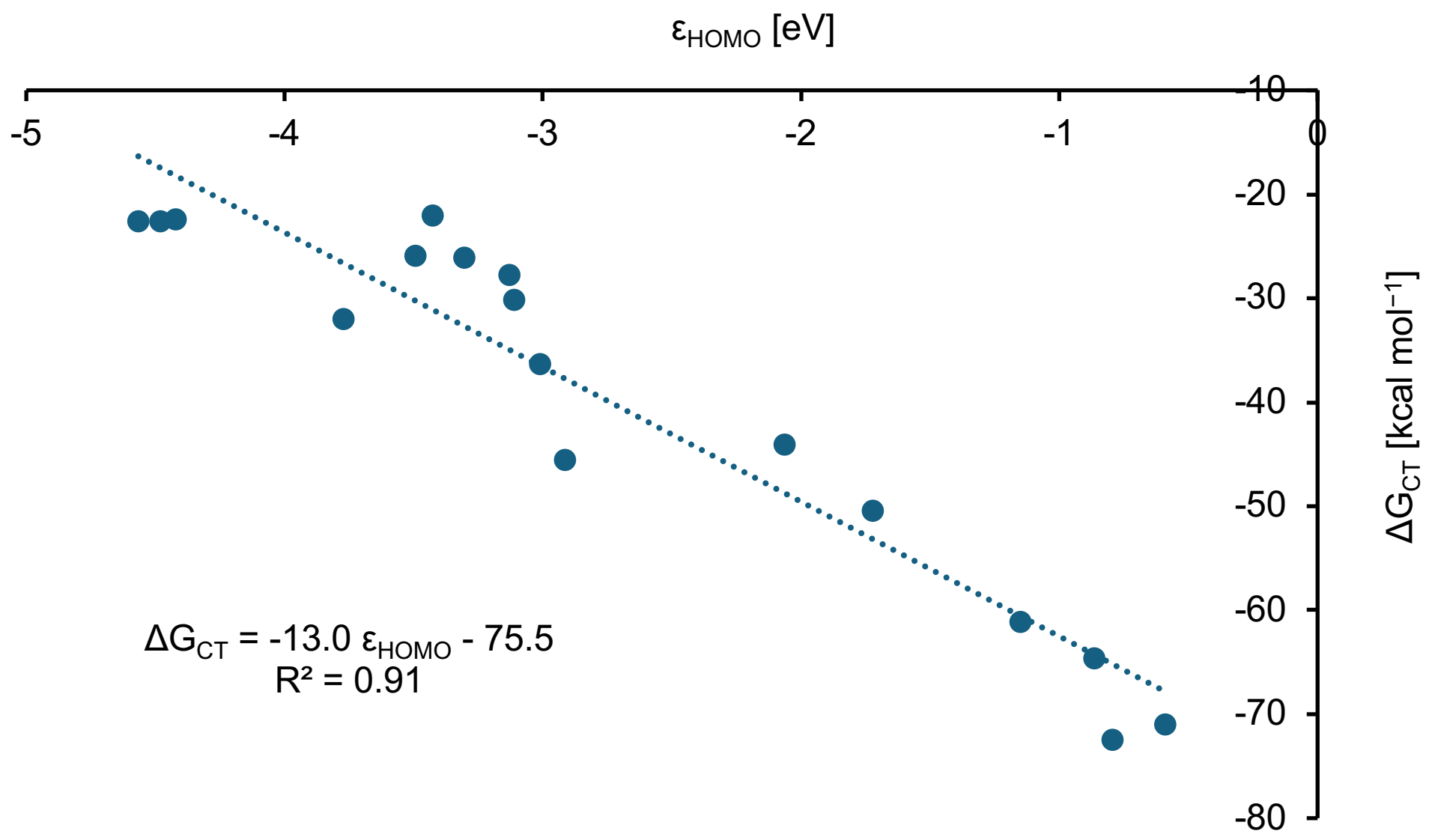

Figure S15 The linear trend between ΔG_{CT} in vacuum and the HOMO energy of all the investigated anions. Replotted based on Figure 4 in main text, using all data points.

Table S16 Formation *electronic energies* of the investigated CT complexes ($Nu^- + I_2 \rightarrow [Nu\text{-}I_2]^-$), the reaction energies for the gross reaction ($Nu^- + I_2 \rightarrow Nu\text{-}I + I^-$), and for the dissociation of the CT complexes ($[Nu\text{-}I_2]^- \rightarrow Nu\text{-}I + I^-$) for nucleophiles Nu^-: maleimide, glutarimide, $[(Me\text{-}CO)_2HC]^-$, $[MeO]^-$, $[MeS]^-$, $[(Me\text{-}CO)\text{-}S]^-$, $[Me_2P]^-$, $[(Me\text{-}CO)_2P]^-$, F^-, Cl^- and Br^- anions. The values were obtained at the **B3LYP-D3BJ/def2-TZVPPD** level of theory both in vacuum and using SMD implicit solvation model and are given in kcal mol^{-1}.

**Nu$^-$**	**ΔE$_{CT}$**		**ΔE$_{rxn}$**		**ΔE$_{diss}$**	
	vacuum	solvent	vacuum	solvent	vacuum	solvent
maleimide	−35.3	−13.7	−3.2	−3.8	+32.1	+9.9
glutarimide	−36.1	−15.9	−8.3	−7.1	+27.8	+8.8
$[(Me\text{-}CO)_2HC]^-$	−37.6	−21.1	−13.8	−17.6	+23.8	+3.5
$[MeO]^-$	−63.6	−25.8	−38.0	−16.7	+25.6	+9.1
$[MeS]^-$	−59.0	−40.8	−39.6	−37.0	+19.4	+3.8
$[(Me\text{-}CO)S]^-$	−45.6	−24.9	−18.4	−19.5	+27.2	+5.4
$[Me_2P]^-$	−87.8	−77.4	−80.5	−76.5	+7.3	+0.9
$[(Me\text{-}CO)_2P]^-$	−45.4	−34.5	−27.5	−33.7	+17.9	+0.8
F^-	−57.1	−13.1	−17.4	+9.0	+39.7	+22.1
Cl^-	−38.7	−12.0	0.2	+7.0	+38.9	+19.0
Br^-	−36.3	−18.7	2.0	−2.6	+38.3	+16.1

Table S17 Formation *Gibbs free energies* of the investigated CT complexes ($Nu^{-} + I_2 \rightarrow [Nu\text{-}I_2]^{-}$), the reaction energies for the gross reaction ($Nu^{-} + I_2 \rightarrow Nu\text{-}I + I^{-}$), and for the dissociation of the CT complexes ($[Nu\text{-}I_2]^{-} \rightarrow Nu\text{-}I + I^{-}$) for nucleophiles Nu^{-}: maleimide, glutarimide, $[(Me\text{-}CO)_2HC]^{-}$, $[MeO]^{-}$, $[MeS]^{-}$, $[(Me\text{-}CO)\text{-}S]^{-}$, $[Me2P]^{-}$, $[(Me\text{-}CO)_2P]^{-}$, F^{-}, Cl^{-} and Br^{-} anions. The values were obtained at the **B3LYP-D3BJ/def2-TZVPPD** level of theory both in vacuum and using SMD implicit solvation model and are given in kcal mol^{-1}.

**Nu⁻**	**ΔG$_{CT}$**		**ΔG$_{rxn}$**		**ΔG$_{diss}$**	
	vacuum	solvent	vacuum	solvent	vacuum	solvent
maleimide	−23.7	−2.5	+0.9	−0.3	+24.6	+2.2
glutarimide	−24.1	−4.1	−3.5	−2.9	+20.6	+1.2
$[(Me\text{-}CO)_2HC]^{-}$	−25.6	−9.2	−8.8	−13.4	+16.8	+4.2
$[MeO]^{-}$	−51.2	−14.9	−32.6	−13.0	+18.6	+1.9
$[MeS]^{-}$	−48.4	−30.4	−36.1	−33.9	+12.3	−3.5
$[(Me\text{-}CO)S]^{-}$	−33.8	−14.3	−14.0	−16.2	+19.8	−1.9
$[Me_2P]^{-}$	−75.3	−66.4	−75.2	−72.2	+0.1	−5.8
$[(Me\text{-}CO)_2P]^{-}$	−33.9	−23.8	−23.4	−30.2	+10.5	−6.3
F^{-}	−49.4	−5.6	−17.1	+9.3	+32.3	+14.9
Cl^{-}	−31.3	−4.7	+0.2	+6.9	+31.5	+11.6
Br^{-}	−28.8	−11.4	+1.8	−2.9	+30.6	+8.5

Table S18 Formation electronic energies *of* the investigated CT complexes (Nu⁻ + I_2 → [Nu-I_2]⁻), the reaction energies for the gross reaction (Nu⁻ + I_2 → Nu-I + I⁻), and for the dissociation of the CT complexes ([Nu-I_2]⁻→ Nu-I + I⁻) for nucleophiles Nu⁻: maleimide*,* glutarimide*,* [(Me-CO)$_2$HC]⁻, [MeO]⁻, [MeS]⁻, [(Me-CO)-S]⁻, [Me2P]⁻*,* [(Me-CO)$_2$P]⁻, F⁻, Cl⁻ and Br⁻ anions. The values were obtained at the **DLPNO-CCSD(T)/def2-TZVPPD**//B3LYP-D3BJ/def2-TZVPPD level of theory both in vacuum and using SMD implicit solvation model and are given in kcal mol^{-1}.

**Nu⁻**	**ΔE$_{CT}$**		**ΔE$_{rxn}$**		**ΔE$_{diss}$**	
	vacuum	solvent	vacuum	solvent	vacuum	solvent
maleimide	−33.8	−12.5	−5.7	−6.9	+28.1	+5.6
glutarimide	−35.1	−15.3	−11.6	−10.5	+24.1	+4.8
[(Me-CO)$_2$HC]⁻	−38.7	−26.5	−21.1	−25.2	+17.6	+1.3
[MeO]⁻	−62.9	−24.3	−41.6	−19.4	+21.2	+4.9
[MeS]⁻	−54.8	−38.9	−39.5	−37.3	+15.3	+1.6
[(Me-CO)S]⁻	−40.2	−20.7	−17.9	−19.5	+22.3	+1.2
[Me$_2$P]⁻	−84.9	−77.0	−79.8	−76.1	+5.0	+0.9
[(Me-CO)$_2$P]⁻	−42.3	−35.2	−27.7	−29.8	+14.6	+5.4
F⁻	−53.8	−9.2	−19.6	+6.8	+34.2	+16.0
Cl⁻	−34.0	−7.2	−4.0	+5.7	+30.0	+12.9
Br⁻	−30.2	−13.8	+1.2	−4.3	+29.0	+9.5

Table S19 Formation *Gibbs free energies* of the investigated CT complexes ($Nu^{-} + I_2 \rightarrow [Nu\text{-}I_2]^{-}$), the reaction energies for the gross reaction ($Nu^{-} + I_2 \rightarrow Nu\text{-}I + I^{-}$), and for the dissociation of the CT complexes ($[Nu\text{-}I_2]^{-} \rightarrow Nu\text{-}I + I^{-}$) for nucleophiles Nu^{-}: maleimide, glutarimide, $[(Me\text{-}CO)_2HC]^{-}$, $[MeO]^{-}$, $[MeS]^{-}$, $[(Me\text{-}CO)\text{-}S]^{-}$, $[Me2P]^{-}$, $[(Me\text{-}CO)_2P]^{-}$, F^{-}, Cl^{-} and Br^{-} anions. The values were obtained at the **DLPNO-CCSD(T)/def2-TZVPPD**//B3LYP-D3BJ/def2-TZVPPD level of theory both in vacuum and using SMD implicit solvation model and are given in kcal mol^{-1}.

**Nu^{-}**	**ΔG_{CT}**		**ΔG_{rxn}**		**ΔG_{diss}**	
	vacuum	solvent	vacuum	solvent	vacuum	solvent
maleimide	−22.2	−1.3	−1.7	−3.2	+20.5	−1.9
glutarimide	−23.1	−3.5	−6.8	−6.3	+16.3	−2.8
$[(Me\text{-}CO)_2HC]^{-}$	−26.8	−14.6	−16.2	−21.0	+10.6	−6.4
$[MeO]^{-}$	−50.4	−13.5	−36.0	−15.7	+14.4	−2.2
$[MeS]^{-}$	−44.1	−28.5	−36.1	−34.3	+8.0	−5.8
$[(Me\text{-}CO)S]^{-}$	−28.4	−10.0	−13.5	−16.3	+14.9	−6.3
$[Me_2P]^{-}$	−72.4	−66.0	−74.5	−71.8	−2.1	−5.8
$[(Me\text{-}CO)_2P]^{-}$	−30.7	−24.6	−23.6	−26.3	+7.1	−1.7
F^{-}	−46.2	−1.7	−19.3	+7.1	+26.9	+8.8
Cl^{-}	−26.5	+0.1	−4.0	+5.5	+22.5	+5.4
Br^{-}	−22.7	−6.5	+1.1	−4.5	+23.8	+1.8

Details of the scan calculations

The source data, i.e. the scanned interatomic distances and the corresponding electronic energies for the 1D and 2D relaxed scan calculations (Figure 3 in main text and Figures S4-S6 and S9-S14 above) are available under 10.5281/zenodo.18911725.

Optimized geometries (in xyz format) and corresponding electronic energies (in a.u)

All the optimized geometries, including stationary points and the corresponding electronic energies in tabular format are provided in a Zenodo directory under 10.5281/zenodo.18911725.
